# Maximizing pancreatic carcinoma classification performance using parrot optimized vision transformer

**DOI:** 10.1038/s41598-026-53240-w

**Published:** 2026-05-21

**Authors:** C. Mallika, E. Dinesh, Hadeel Alsolai, Munya A. Arasi

**Affiliations:** 1 Department of Master of Computer Applications, E.G.S. Pillay Engineering College, Nagapattinam, Tamil Nadu 611002 India; 2 Department of Electronics and Communication Engineering, M. Kumarasamy College of Engineering, Karur, Tamil Nadu 639113 India; 3 Department of Information Systems, College of Computer and Information Sciences, Princess Nourah Bint Abdulrahman University, P.O. Box 84428, 11671 Riyadh, Saudi Arabia; 4 Department of Computer Science, Applied College at Rijal Almaa, King Khalid University, Abha, Saudi Arabia

**Keywords:** Gabor filter, YOLOv11, UNet, Vision transformer and parrot optimizer, Cancer, Computational biology and bioinformatics, Engineering, Mathematics and computing

## Abstract

Pancreatic cancer is a rare kind of cancer that is detected during the final stages. This is because the symptoms are very common and also do not show up in the starting phase. Hence an automated system for identification and classification of pancreatic cancer becomes essential. This becomes possible with the help of artificial intelligence and machine learning. The aim of this research is to develop a model that classifies pancreatic cancer using Pancreatic CT image dataset involving 1411 images from Kaggle website. The input images are augmented for increasing the dataset quality and preprocessed using Gabor filter. Segmentation is performed using UNet and features are extracted using YOLOv11 model. Pancreatic carcinoma classification is achieved using a modern deep learning-based classifier called the Vision Transformer. The classified results are optimized with the help of Parrot metaheuristic optimization algorithm. The proposed model produced an accuracy of 99%, precision of 98.5%, recall value of 97.7%, F1-Score of 96.4% and Matthew’s correlation coefficient value of 97.3% in addition to true positive and false positive rates of 96.1% and 0.07%. These results are considered phenomenal and superior when compared to existing models of Random Forest, Convolutional Neural Network, Deep belief networks, and Support Vector Machine.

## Introduction

Pancreatic cancer remains one of the most aggressive and life-threatening malignancies due to its asymptomatic progression and late-stage diagnosis, leading to extremely poor survival rates^1–4^. Despite continuous advancements in medical imaging and computational intelligence, early detection of pancreatic carcinoma continues to be a significant clinical challenge. Conventional diagnostic approaches rely heavily on radiological expertise, which introduces subjectivity and inter-observer variability. Although artificial intelligence-based techniques have been increasingly adopted for pancreatic cancer diagnosis, many existing models focus on isolated tasks such as segmentation or classification, thereby limiting their ability to capture the complex structural and contextual characteristics present in computed tomography (CT) images^5,6^.

Deep learning-based approaches, particularly Convolutional Neural Networks (CNNs), have demonstrated strong performance in extracting local spatial features. However, these models often struggle to capture long-range dependencies and global contextual relationships within medical images. Recently, transformer-based architectures have emerged as powerful alternatives due to their ability to model global interactions through self-attention mechanisms^7,8^. Nevertheless, transformer models require robust and informative feature representations for optimal performance and are sensitive to variations in input data. Furthermore, most existing studies lack a unified framework that integrates preprocessing, segmentation, feature extraction, and classification in a coherent manner.

Another critical limitation observed in current methodologies is the insufficient integration of detection-based feature extraction techniques with transformer-based classification models. While segmentation architectures such as UNet effectively localize regions of interest^9^, and detection-based models such as YOLO variants provide discriminative multi-scale feature representations^10^, their combined utilization for pancreatic cancer diagnosis remains limited. Additionally, optimization strategies in many studies are confined to conventional training procedures, without incorporating adaptive metaheuristic mechanisms to refine classification outcomes.

Moreover, several existing works report high classification accuracy; however, they often lack comprehensive evaluation using clinically relevant metrics such as Matthew’s correlation coefficient (MCC) and false positive rate (FPR), which are critical for ensuring reliability in medical diagnosis^11,12^. In addition, many approaches are constrained by limited exploration of hybrid architectures that can simultaneously exploit spatial localization, feature abstraction, and global contextual modeling.

Motivated by these challenges, there is a need for a comprehensive and integrated framework that can effectively combine multiple complementary techniques to enhance diagnostic performance. The proposed work addresses this requirement by developing a unified pipeline that incorporates preprocessing, UNet-based segmentation, YOLOv11-based feature extraction, Vision Transformer-based classification, and Parrot Optimization for performance refinement. This integrated approach enables improved representation learning, enhanced classification accuracy, and reduced misclassification, thereby contributing to a more reliable and efficient system for pancreatic cancer diagnosis.

### Problem statement and motivation

The prognosis of pancreatic cancer remains extremely poor due to its late-stage detection and the absence of distinct early symptoms, leading to a significantly low survival rate^3,4^. Although treatment options are available, their effectiveness is highly dependent on early diagnosis, which is often not achieved in clinical practice. Conventional diagnostic procedures primarily rely on radiological interpretation, which may result in variability due to differences in expertise, fatigue, and subjective judgment. This variability can lead to delayed or inaccurate diagnosis, thereby increasing the risk to patient life.

In recent years, artificial intelligence-based approaches have been explored to improve diagnostic accuracy; however, many existing methods focus on single-stage learning and lack the ability to simultaneously capture fine-grained spatial details and global contextual information from CT images^5,6^. Moreover, several models do not incorporate robust feature extraction and optimization mechanisms, which are essential for handling the complexity and variability present in pancreatic imaging data. As a result, there remains a need for a more comprehensive and reliable framework that can address these limitations and support early-stage detection.

Motivated by these challenges, the objective of this research is to develop an integrated deep learning-based system that enhances the accuracy and consistency of pancreatic cancer classification. By combining advanced segmentation, feature extraction, transformer-based learning, and optimization techniques, the proposed approach aims to reduce diagnostic variability and improve decision support for clinicians. Such a system has the potential to assist medical practitioners in making more informed and consistent decisions, thereby contributing to improved patient outcomes and more effective management of pancreatic carcinoma.

### Novelty of proposed work

The proposed work presents a novel and integrated framework for pancreatic cancer classification by combining multiple complementary deep learning components into a unified pipeline. Unlike existing approaches that predominantly rely on standalone convolutional or transformer-based models, the proposed system effectively bridges the gap between spatial localization and global contextual learning by integrating UNet-based segmentation^9^, YOLOv11-driven feature extraction^10^, and vision transformer-based classification^13^.

A key novelty of this work lies in the utilization of a detection-oriented feature extraction mechanism prior to transformer-based classification. While UNet accurately isolates the region of interest, the incorporation of YOLOv11 enables extraction of discriminative multi-scale features, which are then effectively modeled using the global attention mechanism of the Vision Transformer. This sequential integration ensures that both local structural details and long-range dependencies are captured efficiently, which is often not achieved in conventional single-stage models.

Another significant contribution is the introduction of the Parrot Optimization algorithm as a post-classification refinement strategy^14^. Unlike traditional optimization approaches that are limited to parameter tuning during training, the proposed method employs a metaheuristic optimization mechanism to enhance classification outcomes, thereby improving robustness and reducing misclassification. This adaptive optimization layer adds an additional level of performance enhancement that is not commonly explored in pancreatic cancer diagnosis frameworks.

Further, the proposed framework emphasizes a balanced and clinically relevant evaluation by incorporating performance metrics such as Matthew’s correlation coefficient (MCC) and false positive rate (FPR), which are crucial for assessing reliability in medical decision-making^11,12^. The structured integration of segmentation, detection-based feature extraction, transformer-based classification, and metaheuristic optimization distinguishes the proposed work from existing methods and contributes to improved diagnostic performance.

### Contribution

This research develops a novel model for identifying and classifying pancreatic cancer using CT images by applying state of the art deep learning-based techniques. The major contributions of this work are stated below.

The major contributions of this research are outlined as follows:

- A unified deep learning framework is developed for pancreatic cancer classification that integrates preprocessing, segmentation, feature extraction, classification, and optimization into a single pipeline.
- UNet-based segmentation is employed to accurately isolate pancreatic regions from CT images, ensuring that relevant structural information is preserved for subsequent processing.
- YOLOv11-based feature extraction is incorporated to obtain discriminative multi-scale features, enhancing the representation capability beyond conventional feature extraction methods.
- A vision transformer-based classifier is utilized to model global contextual dependencies among image patches, improving classification performance compared to traditional convolution-based approaches.
- A parrot optimization algorithm is introduced as a post-classification refinement mechanism to enhance classification accuracy and reduce misclassification rates.
- Comprehensive evaluation is performed using multiple performance metrics including accuracy, precision, recall, F1-score, and Matthew’s correlation coefficient (MCC), along with comparative analysis against conventional classifiers.

## Related works

The research presented in^15^ discusses about the different applications of deep learning in the diagnosis and treatment of pancreatic cancer. The application of artificial intelligence and its related techniques in the healthcare industry have been increasing year by year for the betterment of patients and their treatment. Apart from this, it also helps in improving the diagnosis accuracy, treatment outcome, and management of medical administration to some extent. A recent survey has shown that the number of publications related to pancreatic diseases in combination with artificial intelligence and deep learning techniques has increased exponentially. Different types of articles such as retrospective, reviews and prospective articles which are based on the PDAC pathology have been discussed with various artificial intelligence algorithms such as convolutional neural network, ResNet50, VGG11, long short term memory, ResNet18, natural language processing, generative adversarial networks, 3D UNet, deep neural networks etc. along with corresponding accuracies.

The authors of^11^ have developed a computer aided diagnosis system for the identification of pancreatic cancer with the help of an automated deep learning model. It has been stated that due to poor prognosis of pancreatic cancer, an intervention is needed which is provided rightly by deep learning techniques. It can fasten the treatment process, reduce the overhead of the medical professional, and help them with more precise and individualized treatment. The biggest advantage of artificial intelligence tools is that they are affordable and easily accessible. It can also be customized according to the need and field in which they are being applied. The application of deep learning-based techniques for the interpretation of medical images has grown prominently because of the promising results that they have been producing over time. CT images which were used as input were classified into 5 stages such as normal, pancreatic tumor, benign, premalignant, and malignant. Augmentation and preprocessing techniques such as vertical and horizontal flipping, resizing and anisotropic diffusion filtering were used. Watershed segmentation and UNet based feature extraction were performed later. An 11 layer of AlexNet CNN model was used for the classification purpose. The images were morphologically refined for extracting features and Grey Level Co-Occurrence Matrix was performed along with texture analysis. The accuracy produced at the end was 99.64% with AUC of 0.9979. The processing time taken by the proposed model was around 1.51 s.

Article^16^ provides a comparative study of random forest and logistic regression in the classification of pancreatic cancer. A real time dataset was collected from Al-Islam hospital in Indonesia which contained 203 patient details including 6 disease attributes such as age, CA 19–9, hemoglobin, leukocyte, hematocrit, thrombosis etc. The data samples were classified using logistic regression and random forest with the help of threefold cross validation technique. The final results show that logistic regression produced an accuracy of 96.48% and random forest classifier produced 99.38% accuracy stating the superiority of random forest classifier.

The findings of^17^ elaborate about the performance of Convolutional Neural Networks (CNN)in the process of grading pancreatic cancer using pathological images.138 pathological images with three different resolutions were obtained. They were stained with May-Grunwald-Giemsa (MGG) and Haematoxylin and Eosin (H&E) stains. Preprocessing was done with squared slicing method. The images were augmented and normalized for obtaining better results. 14 CNN models such as VGG16, VGG19, Xception, ResNet50, InceptionV3, Mobile net, DenseNet121, NasNet and more were used employed for classification. The results were fine-tuned and in the end, it was DenseNet201 that achieved highest F1-scores of 0.8786, 0.9561 and 0.8915 respectively for MGG, H&E and mixed stains.

The authors of^12^ have presented a research article regarding the classification of gastrointestinal cancer with the help of CapsNet and Deep Belief Network (DBN). The Kvasir data set containing 5000 samples were used for execution of the proposed model and the images were preprocessed using bilateral filtering. Feature extraction was done using CapsNet and classification was performed by a deep belief network-based classifier. Snake optimization algorithm (SOA) was used for improving the classification results which led to a classification accuracy of 99.72%.

The research findings in^18^ elaborate s about the classification of pancreatic cancer into three different types such as chronic pancreatitis, intraductal papillary mucinous neo plasm and pancreatic carcinoma using two novel support vector machine-based classifiers. Stable nested SVM and margin moment based SVM are the two types of classifiers proposed in this article. For the nested SVM, the training accuracy was 81.82% and for the moment based SVM classifier the classification accuracy was 66.82%.

Article^19^ has proposed a novel Vision transformer based pancreatic cancer classification system using shuffle instance and Rapid On-Site Evaluation (ROSE) technique. For the execution of the proposed model real time ROSE images were acquired from Peking Union Medical College hospital with the approval of the Ethics Committee. The data was then labeled and preprocessed using bag of patches which were then split and labels were assigned. For the classification purpose, a vision transformer and two multilayer perceptron-based heads were used. A shuffle step is performed in order to create bags of shuffle instances for better understanding of the relationships among the input samples. Finally, a classification accuracy of 94% was achieved by the proposed classifier with a precision value of 91.98% and recall value of 90.68%.

A robust DACTransNet has been proposed in^20^ for the classification of histopathological images of pancreatic cancer. Spatial pyramids have been appended to the architecture for more accurate results and automation. Features have been extracted using Convolutional Neural networks which extract both abstract and spatial features. Migration learning was applied at the end for bettering the obtained classification accuracy of 96%. Table 1 lists the important details of related articles that have been discussed in this section.

**Table 1:** Analysis of related works.

Author	Year	Dataset	Method	Results
H Patel et al	2024	–	ResNet50, VGG11	–
A Nadeem et al	2025	Pancreatic CT images	AlexNet	Accuracy—99.64%
Zuherman Rustam et al	2021	Real time dataset	Logistic regression, Random forest	Accuracy—99.38%
Muhammad Nurmahir Mohamad Sehmi et al	2022	Real time dataset	Convolution Neural Networks	F1-Score—0.9561
FA Almarshad et al	2024	Kvasir dataset	Deep Belief Network	Accuracy—99.72%
Ammon Washburn et al	2023	Karyometry data	Support Vector Machine	Accuracy—66.82%
Tianyi Zhang et al	2023	Real time dataset	Vision transformer	Accuracy—94%
Yongqing Kou et al	2024	–	DACTransNet	Accuracy—96%

From the critical analysis of existing studies^21,22^, it is observed that most pancreatic cancer diagnosis frameworks have primarily focused on either conventional machine learning classifiers, CNN-based models, or standalone transformer architectures. Although these methods have produced encouraging results, several limitations remain. First, many works emphasize final classification accuracy without sufficiently explaining how region localization, feature representation, and classification decisions are connected. Second, several CNN-based approaches are effective in extracting local features but have limited ability to model long-range contextual relationships within CT images. Third, transformer-based models can capture global dependencies, but their performance depends strongly on the quality of input feature representations. Fourth, optimization strategies in existing studies are mostly restricted to standard training procedures and do not provide an additional adaptive refinement mechanism for improving classification outcomes.

The reviewed works also indicate that the integration of segmentation, detection-oriented feature extraction, transformer-based classification, and metaheuristic optimization has not been adequately explored for pancreatic CT image classification. For instance, UNet-based approaches support region-level segmentation, while YOLO-based models provide strong multi-scale feature representation. However, their combined use with Vision Transformer classification remains limited. Similarly, although optimization algorithms have been applied in some medical image classification studies, their role in refining transformer-based pancreatic cancer classification has not been sufficiently examined. These gaps show the need for a unified framework that can combine local structural information, multi-scale discriminative features, global contextual learning, and adaptive optimization.

The proposed work addresses these limitations by developing an integrated ViT-PO framework. In this model, Gabor filtering enhances the CT images before segmentation, UNet isolates relevant pancreatic regions, YOLOv11 extracts discriminative multi-scale features, and Vision Transformer captures global dependencies for classification. The Parrot Optimization algorithm is then incorporated to refine the classification performance. Therefore, instead of merely applying a single classifier, the proposed framework establishes a sequential and complementary pipeline in which each stage supports the next stage. This structured integration directly addresses the gaps identified in previous works and improves the reliability of pancreatic carcinoma classification.

## Proposed methodology

**Fig. 1:**
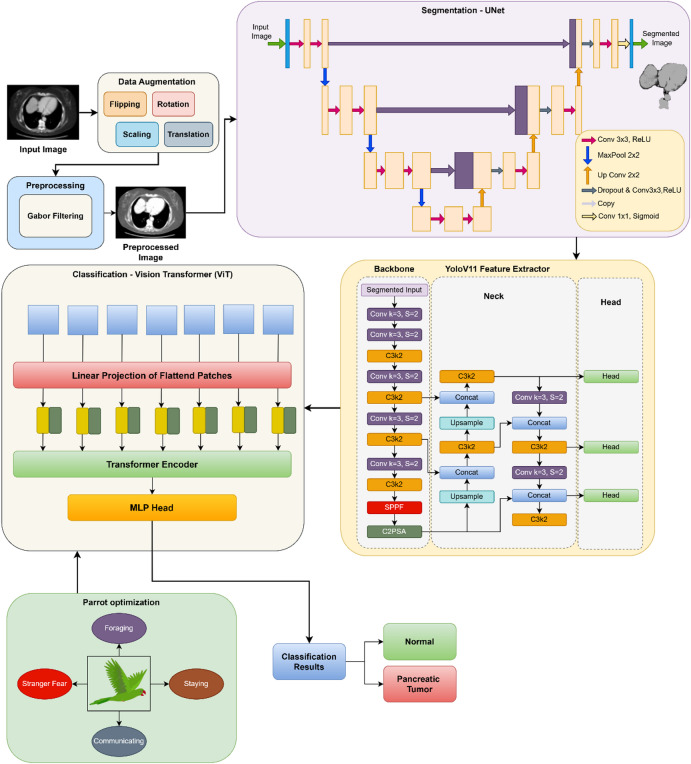
Developed ViT-PO Model.

The proposed vision transformer based parrot optimization (ViT-PO) model has been developed with an aim to classify pancreatic cancer images with the help of computed tomography (CT) images utilizing the power of deep learning and artificial intelligence. The input images were obtained from Kaggle website named Pancreatic CT image dataset. It comprises 1411 CT images which were divided into a ratio of 70:30 for the purpose of training and testing. Data augmentation is performed on the input images using methods like rotation, horizontal flipping, vertical flipping, scaling, and translation. After augmenting the images, they are preprocessed using Gabor filter. UNet based segmentation is done for segmenting the preprocessed CT images, after which feature extraction is performed with the help of YOLOv11 model. From the extracted features, pancreatic carcinoma classification is accomplished into two classes such as normal and pancreatic tumour using Vision Transformer based classifier. Parrot optimization algorithm is then applied on the acquired results for further improving the classification accuracy. Performance of the proposed vision transformer based pancreatic cancer classification system is evaluated by calculating metrics of accuracy, precision, recall, F1-Score and Matthew’s correlation coefficient (MCC). Once the performance has been evaluated, they are compared with existing classifiers like Random Forest, Convolutional Neural Network, Deep Belief Networks and Support Vector Machine which will yield a comparative analysis for projecting the outstanding performance of the proposed model. Figure 1 shows the workflow of the proposed ViT-PO based pancreatic cancer classification.

The input CT image is first preprocessed using Gabor filtering and then passed to the UNet model to obtain a segmentation mask. This mask is applied to the original image to generate a region-focused (masked) image, which serves as the input to the YOLOv11 backbone. YOLOv11 then performs feature extraction, producing compact multi-scale feature representations. These extracted features are subsequently converted into patch embeddings and provided as input to the Vision Transformer, which performs the final classification. Thus, the pipeline follows a clear flow: Preprocessing→Segmentation (UNet)→Masked Image Generation→Feature Extraction (YOLOv11)→Patch Embedding→Classification (ViT) →Optimization (POA). No parallel or ambiguous feature paths are involved.

### Data augmentation

Data augmentation is the process of creating new images that are similar to the input imagesfor inflating the dataset. This is performed primarily for enhancing the total number of images that are about to be used for execution of the proposed system. It is done by multiplying the existing images with methods like normalization, rotation, horizontal flipping, vertical flipping, scaling and translation^23^. Equations for the methods are given below in (1) to (6).

1 $$D_{{{\mathrm{nor}}}} \left( {k,l} \right) = \frac{{D\left( {k,l} \right) - \min (D)}}{\max \left( D \right) - \min (D)}$$

where D—input image, min(D) and max(D) represent the minimum and maximum values of pixel, ${D}_{nor}$ indicates the normalized image.

2 $$D_{{{\mathrm{rot}}}} \left( {k,l} \right) = D_{{{\mathrm{nor}}}} (k\,\cos \theta - l\,\sin \theta , k\,\sin \theta + l\,\cos \theta )$$

3 $$D_{{{\mathrm{hor}}}} \left( {k,l} \right) = D_{{{\mathrm{nor}}}} (k, Y - l - 1)$$

4 $$D_{{{\mathrm{ver}}}} \left( {k,l} \right) = D_{{{\mathrm{nor}}}} (X - k - 1,l)$$

5 $$D_{{{\mathrm{sca}}}} \left( {k,l} \right) = D_{{{\mathrm{nor}}}} \left( {\frac{k}{s},\frac{l}{s}} \right)$$

6 $$D_{{{\mathrm{tra}}}} \left( {k,l} \right) = D_{{{\mathrm{nor}}}} (k + \Delta k, l + \Delta l)$$

Here ${D}_{rot}\left(k\mathrm{,}l\right)$ stands for rotation process, ${D}_{{\mathrm{h}}or}\left(k\mathrm{,}l\right)$ for horizontal flipping, ${D}_{ver}\left(k\mathrm{,}l\right)$ for vertical flipping, ${D}_{sca}\left(k\mathrm{,}l\right)$ for scaling and ${D}_{tra}\left(k\mathrm{,}l\right)$ for translation tasks.

### Preprocessing with Gabor filter

Medical images need to be dealt with extreme care because each and every pixel is absolutely essential. But noisy and unwanted information must be eliminated as well for easier and precise processing. In this case, a Gabor filter is employed for achieving preprocessed inputs. Basically, categorized as a linear, orientation sensitive filter, it is the combination of sinusoidal wave and gaussian functions^24^. They have been applied to a variety of computer vision related tasks such as preprocessing, edge detection, segmentation, object detection etc. It can obtain good resolutions in both spatial and frequency domains. It is two dimensional in form consisting of several rotations, convolution functions, and dilations. A simple product of gaussian kernel along with the sinusoidal signal gives the result of this filter. Its impulse response is defined by the Fourier transformation of both the products. It is also a bandpass filter which has the ability to permit certain signals and forbid unwanted noisy signals. The function of Gabor filter can be represented by factors such as wavelength, phase offset value, frequency, orientation, standard deviation of the gaussian kernel. The equation for the representation of Gabor filter is given below in Eq. (7).

7 $$gf\left( {a,b} \right) = p\left( {a,b} \right)q_{r} (a,b)$$

$p\left(a\mathrm{,}b\right)$ represents the sinusoidal wave as defined in Eq. (8) and ${q}_{r}\mathrm{(}a\mathrm{,}b\mathrm{)}$ indicates the gaussian function.

8 $$p\left( {a,b} \right) = \exp \left( {i\left( {2\pi \left( {m_{0} a + n_{0} b} \right) + {\mathrm{SP}}} \right)} \right)$$

${\mathrm{(}m}_{0}\text{, }{n}_{0}\mathrm{)}$ stands for the frequency and SP is the phase value. It has certain parts associated with it such as the real and imaginary parts which can be seen in (9) and (10).

9 $${\mathrm{real}}\left( {gf\left( {a,b} \right)} \right) = \cos \left( {2\pi (m_{0} a + n_{0} b) + {\mathrm{SP}}} \right)$$

10 $${\mathrm{imag}}\left( {gf\left( {a,b} \right)} \right) = \sin \left( {2\pi (m_{0} a + n_{0} b) + {\mathrm{SP}}} \right)$$

The magnitude (${M}_{0}$) and direction (${\omega}_{0}$) are given by Eqs. (11) and (12) as shown below.

11 $$M_{0} = \sqrt {m_{0}^{2} + n_{0}^{2} }$$

12 $$\omega_{0} = \tan^{ - 1} \left( {\frac{{n_{0} }}{{m_{0} }}} \right)$$

Gabor filtering is employed in this work due to its strong capability in capturing both spatial and frequency domain information, which is particularly important for medical CT images where subtle texture variations indicate pathological changes. Unlike conventional smoothing or denoising filters (e.g., Gaussian or median filters) that primarily suppress noise, Gabor filters are orientation- and frequency-selective, enabling them to enhance edge, boundary, and texture-specific features relevant to pancreatic tumour regions^24^.

Compared to its alternatives, Gabor filtering provides superior localization in both spatial and frequency domains, allowing better preservation of fine structural details while reducing noise. This makes it more effective for preprocessing in segmentation and feature extraction pipelines, as it improves the quality of input data for subsequent UNet segmentation and YOLOv11 feature extraction stages.

### UNet based segmentation

Segmentation is done by UNet architecture of convolutional neural network. It is a very precise model that has been built and refined iteratively for performing image processing tasks such as segmentation and so on. It gains the name from its U-shaped symmetrical architecture which is made up of several encoders and decoders. It takes very little processing time even for segmenting large scale input images and can perform well with a tiny dataset. It is because of these two reasons that they are overwhelmingly used in medical image processing. The key components of a UNet model are the encoder set, decoder set and bottleneck layer in between both of them^9^.

The encoding block consists of a 2*2 max pooling layer and Rectified Linear Unit (ReLu) functions to impart no linearity for enhanced processing. Decoding block comprises layers of upsampling, 3*3 convolutions and skip connections. Upsampling is performed to regain the image size convolution is done in order to optimize the output. Skip connections meanwhile help to retrieve any lost information in the whole process. Bottleneck layer contains the fine-grained information that acts as the core part of the entire model. The architecture has been designed in such a manner that at each level of encoding, the size is reduced while retaining the key features and at the decoding end, the actual image is recreated by swift mapping employing skip connections. The energy function of the model is defined by the Eq. (13)

13 $$C_{f} = \mathop \sum \limits_{p = 1}^{N} s\left( t \right)\log \left( {d_{l\left( t \right)} \left( t \right)} \right)$$

where ${C}_{f}$ shows the energy function of the channel, $t$ represents the features, $s$ indicates the weight, ${d}_{l}$ shows the final feature map which is further defined in (14).

14 $$d_{l} = \exp \left( {{\mathrm{act}}_{l} \left( t \right)} \right)/\mathop \sum \limits_{{l^{\prime} = 1}}^{L} \exp \left( {{\mathrm{act}}_{l} \left( t \right){\prime} } \right)$$

**Fig. 2:**
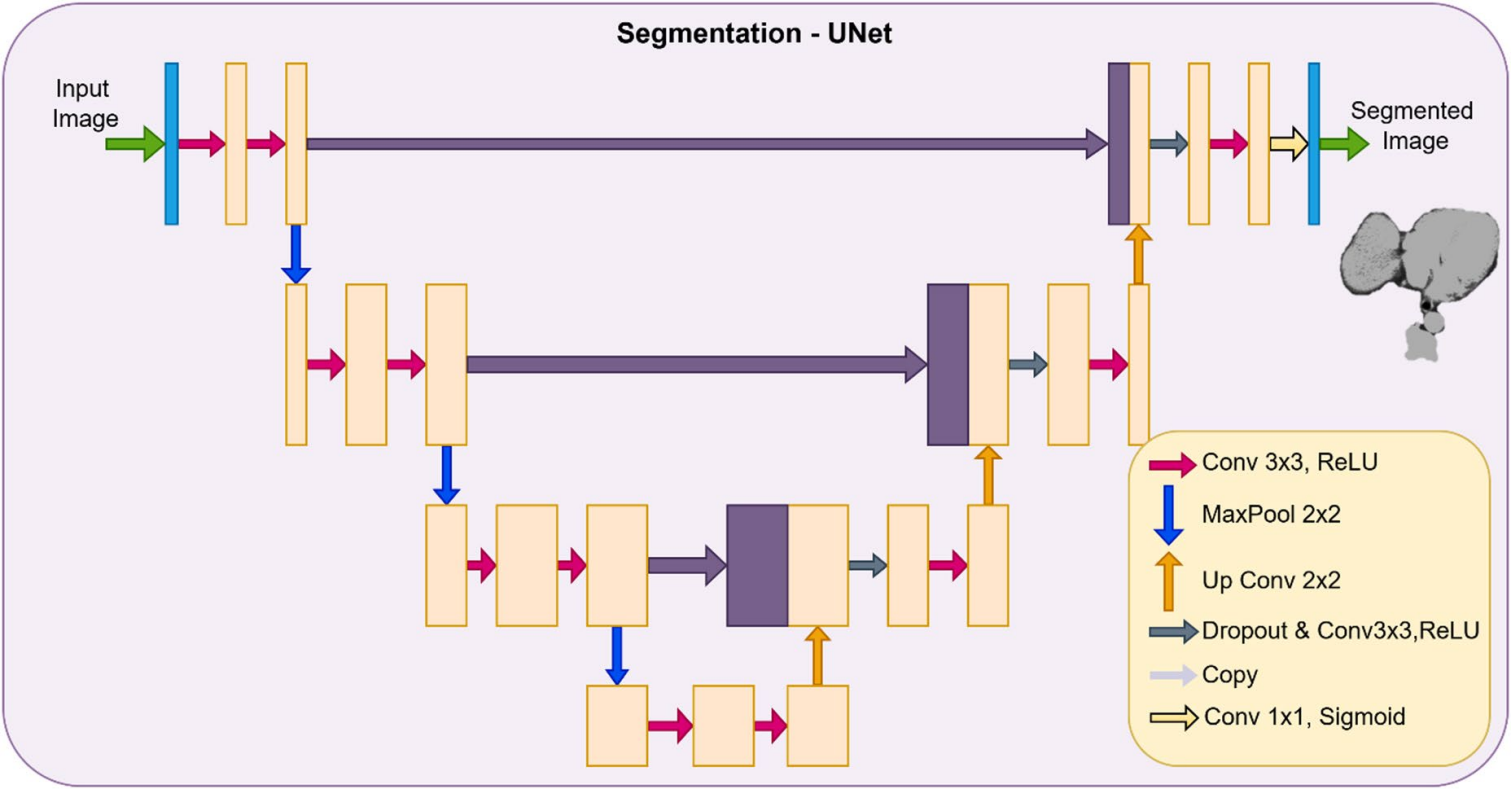
Architecture of UNet.

L is the channel and ${act}_{l}$ is channel activation. Figure 2 below depicts the architecture of UNet.

### Feature extraction using YOLOv11

You only look once (YOLO) is a very successful model for object detection, feature extraction, and localization. It has been elegantly designed in such a way to take up a wide range of image processing tasks. Its diverse nature makes it easily adaptable and compatible with many other existing models. The key advantages of this model are elevated accuracy, better tradeoff and applicability, enhanced object detection, optimized accuracy etc. The specific advancements to this latest version of YOLOv11 are the addition of new blocks for enhanced performance such as C3K2 block, Spatial Pyramid Pooling Fast (SPPF) block, Cross Stage Partial with Spatial Attention block (C2PSA).

In the proposed framework, YOLOv11 is utilized to extract high-level discriminative and multi-scale spatial features from CT images. These features include region-aware representations such as object boundaries, intensity variations, texture patterns, and spatial localization cues that are critical for distinguishing between normal and pancreatic tumour regions. The backbone of YOLO captures hierarchical convolutional features, while its neck (with SPPF and attention blocks) enhances multi-scale feature aggregation, resulting in rich and compact feature embeddings suitable for downstream classification^10^.

The use of YOLO is motivated by its ability to efficiently learn localized and context-aware feature representations, unlike traditional feature extractors that may only capture global or handcrafted descriptors. This makes it particularly effective in medical imaging tasks where subtle structural variations need to be identified.

In this work, feature extraction is performed on the segmented regions obtained from the UNet model, rather than on the entire image. This ensures that the extracted features are focused specifically on the relevant pancreatic region, reducing background noise and improving the quality and relevance of the feature representations. This targeted feature extraction directly contributes to improved classification performance when passed to the Vision Transformer.

Three basic components that make up the architecture of YOLO are the head, neck, and backbone^10^. The backbone is the most crucial block of the YOLO architecture as it acts as the chief feature extractor. It performs tasks such as convolutions, batch normalization, max pooling, and concatenation with the help of sub-blocks like C3K2 and bottle neck layer. Equation (15) gives the operation of standard convolution.

15 $$c\left( {{\mathrm{pos}}_{0} } \right) = \mathop \sum \limits_{{{\mathrm{pos}}_{n \in R} }} w({\mathrm{pos}}_{n} ) \cdot i({\mathrm{pos}}_{0} + {\mathrm{pos}}_{n} )$$

Here, *c* stands for output value at position ${pos}_{0}$, $R$ represents the sample points, ${pos}_{n}$ shows the offset value at the selected position, *w* indicates the kernel weight and *i* is the input map value.

The small 3*3 kernels present in the C3K2 block allows extraction of minor features from the image. The neck region is yet another crucial block which collects the information from the backbone and passes it on to the head. It collects features with the help of the newly added SPPF block using repeated max pooling operations. Similarly, the C2PSA block brings attention mechanisms into this architecture for concentration on specific regions of interest. Upsampling happens here in multiple scales for concatenating and combining the features obtained so far.

**Fig. 3:**
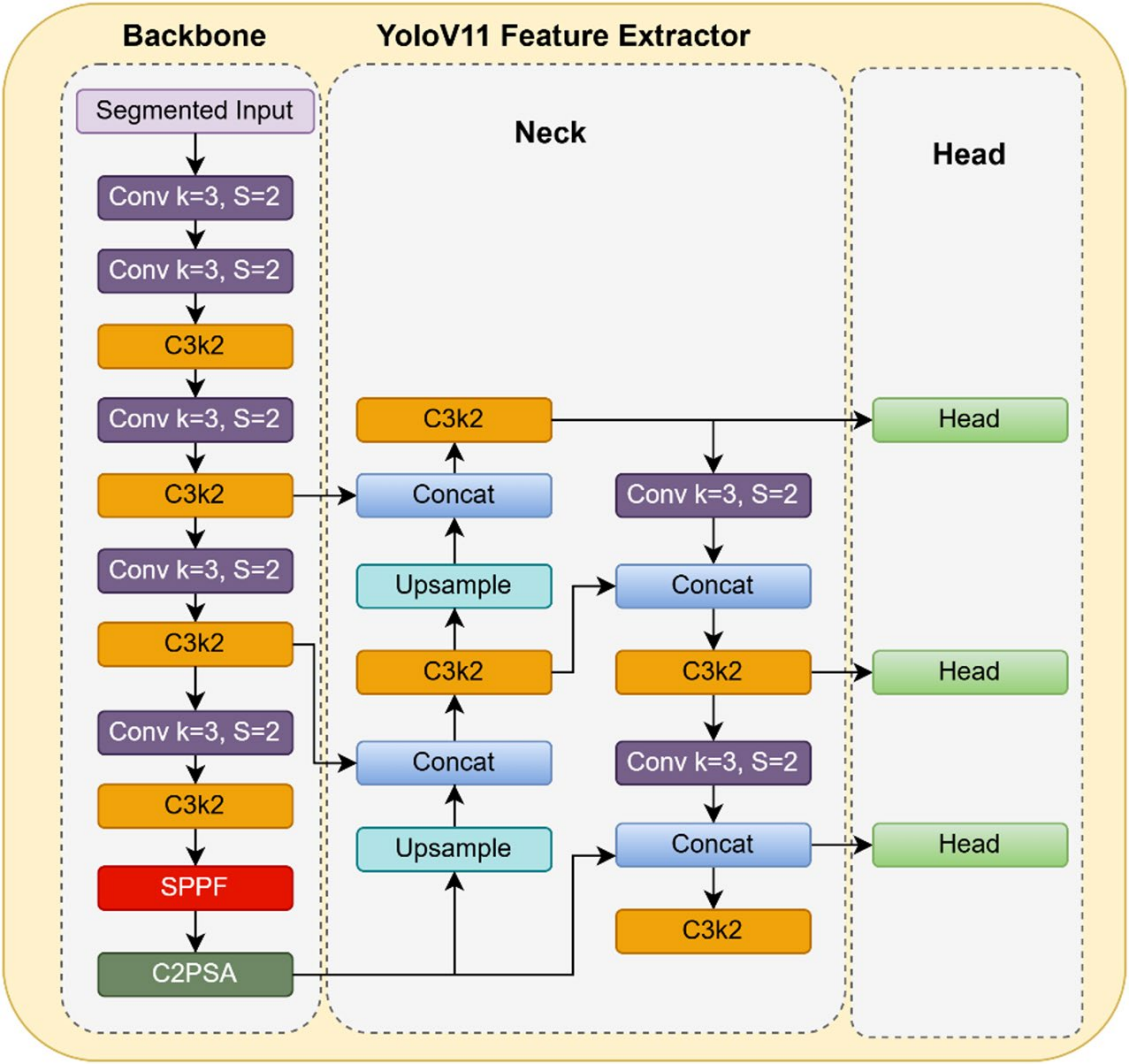
Architectural model of YOLOv11.

The head block consists of multiple levels of detection boxes namely low, medium, and high referring to the different stages and resolutions of the image. CBS (Convolution Batch-norm SiLu layers) block is also present in the head region which refines the output produced by the neck and backbone regions. Figure 3 below represents the architecture of YOLOv11 highlighting the vital components.

In the proposed framework, the output of the UNet segmentation is not used as a separate parallel input but is directly integrated into the feature extraction stage through region-focused masking. Specifically, the segmentation mask produced by UNet is applied to the original CT image to isolate the pancreatic region, effectively suppressing background and irrelevant structures. This masked (segmented) image is then provided as the input to the YOLOv11 backbone for feature extraction. Thus, YOLOv11 operates on region-refined inputs, ensuring that the extracted features are concentrated on clinically relevant areas rather than the entire image. This sequential integration establishes a clear dependency where segmentation enhances feature quality by reducing noise and improving spatial focus.

### Classification using vision transformer

Vision transformer (ViT) is a novel deep learning transformer model that has been adapted specially for computer vision tasks. Basically, used in natural language processing applications, ViTs are now available for use in several image-based classification tasks and have emerged as the opponent of convolutional neural network (CNN). It divides the image into patch sequences and then converts them into vectors and subsequent matrices. Its architectural innovations have led to such tremendous performance. Instead of convolutions, it uses self-attention methods for processing spatial relations among the images. ViTs have developed a strong space for themselves in the medical imaging sector where their ability can span from medical image segmentation, classification, object detection to image synthesis, generation, and restoration. The key advantages of this model over CNN are the large data handling capacity, ability to extract global dependencies, which is highly flexible due to the absence of convolutions and improved end accuracy in the classification tasks. They also suffer from certain limitations like more training time required and also computational expenses sometimes.

There are several computational steps involved in the architecture of a vision transformer such as dividing the images into patches, flattening and embedding them^13^. The next step is positional encoding, adding tokens and passing through layers of encoders. Finally, classification is performed using a MultiLayer Perceptron (MLP) head.

*Step 1*: Patch division

Instead of treating an image as a pixel grid, a vision transformer treats it as a patch sequence. Hence the first step is to divide the images into subsequent fixed sized patches which do not overlap with each other. The created patches are then flattened into single dimension vectors. Equation 16 describes this step.

16 $$r_{0} = \left[ {c_{{{\mathrm{class}}}} ;c_{p}^{1} E;c_{p}^{2} E; \ldots .;c_{p}^{N} E} \right] + E_{{{\mathrm{posi}}}}$$

17 $$E \in R^{{(P^{2} \cdot C)*D}}$$

18 $$E_{{{\mathrm{posi}}}} \in R^{{\left( {N + D1} \right)*}}$$

Here* p* is a predefined value,* n* is the line vector, *r* is the result, *E* stands for the embedding tensor.

*Step 2*: Embedding patches

The flattened patches are then embedded using a linear projection which lets them learn more robust features from the image.

*Step 3*: Encoding of positions

The resulting patch embeddings are then augmented with positional information for better understanding of relative positions among the input image. This is done to keep track of the patch positions and retain the spatial order. A token is then appended to each patch embedding for collective information gathering.

*Step 4*: Transformer encoders

**Fig. 4:**
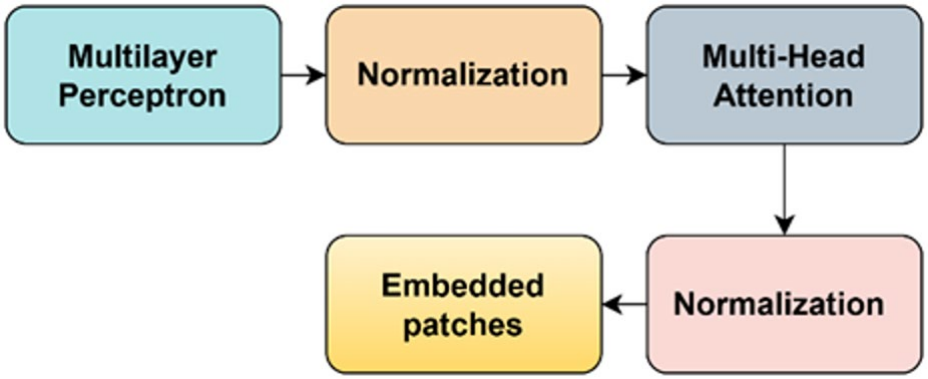
Layers of transformer encoder.

After patches are created and their positional information added to them, they are put through a set of transformer encoder layers which consist of Multi-head Self-attention (MSA). MSA refers to the process of self-attention carried out in multiple heads which enables to aggregate rich information across all directions. Once the self-attention process is complete, patches are again passed into a Feed Forward Network (FFN). The brain behind vision transformers is the self-attention mechanism which is nothing but an entity that is used to compute the interactions and dependencies present in the image patches and thereby learn the hierarchical relationships amongst them as given in Eqs. (19) to (26).

19 $$sa = {\mathrm{soft}}\max (qk^{T} /\sqrt {D)}$$

20 $$sa \in R^{n*n}$$

21 $$r_{l}{\prime} = {\mathrm{MSA}}\left( {{\mathrm{LN}}\left( {r_{l - 1} } \right)} \right) + r_{l - 1}$$

22 $$r_{l} = {\mathrm{MLP}}\left( {{\mathrm{LN}}\left( {r_{l}{\prime} } \right)} \right) + r_{l}{\prime}$$

where l = 1……..L, L represents the transformer stack.

23 $$\left[ {q,k,v} \right] = rW_{qkv}$$

24 $$W_{qkv} \in R^{M*3M}$$

25 $$sa\left( r \right) = sa_{l} \cdot v$$

26 $${\mathrm{MSA}}(r) = \left[ {sa_{1} \left( r \right);sa_{2} \left( r \right); \ldots ..;sa_{b} (r)} \right] + W_{{{\mathrm{msa}}}}$$

q stands for query, k for key and v for value. Similarly, b stands the number of heads, D is the dimension. Figure 4 shows the layer details of transformer encoder.

*Step 5*: Classification head

**Fig. 5:**
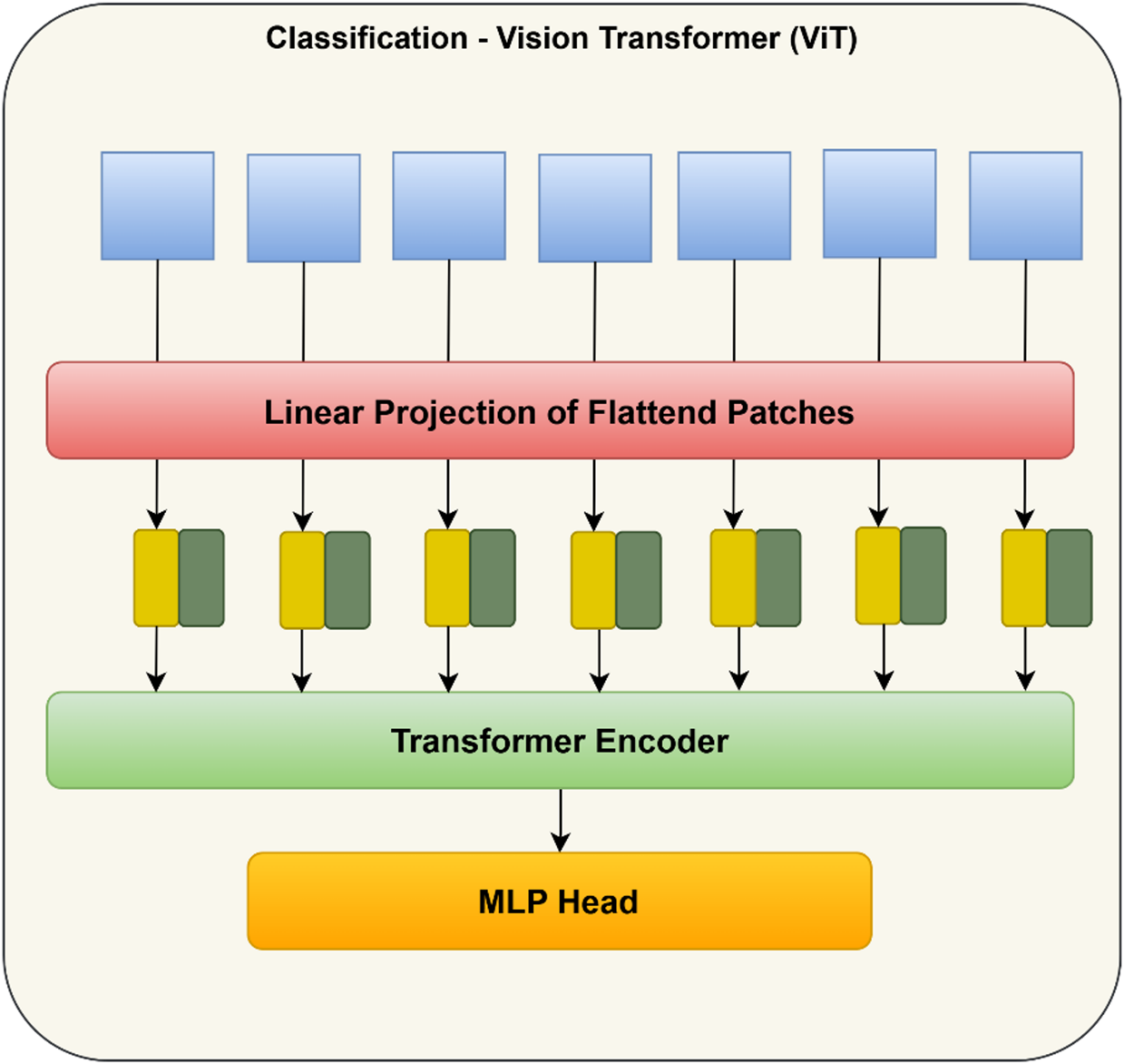
Vision transformer architecture.

The output of the transformer encoders and FFN is used for the classification purpose and given as input to a MLP which typically contains fully connected layers and a softmax layer at the end. Figure 5 shows the depiction of ViT architecture.

### Parrot optimization algorithm

The parrot optimization algorithm (POA) is employed in this work to enhance the classification performance by providing an additional adaptive optimization layer beyond conventional training-based optimization. While the Vision Transformer is trained using standard optimizers such as Adam, POA is applied as a refinement mechanism to further improve the classification outcomes by exploring the solution space more effectively^14^.

POA belongs to the class of population-based metaheuristic algorithms and its counterparts include widely used techniques such as genetic algorithm (GA), particle swarm optimization (PSO), Harris Hawk optimization (HHO), and red deer optimization (RDO). Compared to these methods, POA offers a balanced exploration–exploitation strategy through its unique behavioral modeling of foraging, staying, communication, and predator-avoidance mechanisms. This enables the algorithm to avoid premature convergence and improves its ability to identify near-optimal solutions in complex, non-linear search spaces.

The key advantage of POA in this work is its ability to adaptively refine classification decisions, thereby reducing misclassification and improving robustness. As demonstrated in the experimental results, the integration of POA leads to improved accuracy compared to other optimization algorithms, highlighting its effectiveness in fine-tuning the classification process. The impact of POA is reflected in the overall performance improvement, particularly in achieving higher accuracy and reduced error rates, making the proposed framework more reliable for medical diagnosis.

Optimization algorithms whose aim is to find fittest solution to the given problem in all aspects have become increasingly important because of the performance trade-offs they provide. They can produce better results, optimize time and valuable resources, and can be widely adapted and applied to both engineering and real-world challenges. Out of the many types of optimization algorithms such as deterministic, metaheuristic and biological algorithms, population inspired biological based evolutionary algorithms have been gaining popularity in recent times. Parrot optimization algorithm is one such metaheuristic algorithm which has been designed based upon the characteristics of parrots belonging to the class Pyrrhura Molinae. Commonly called green cheeked parrots, they are extensively domesticated and have certain unique behaviors such as foraging habits, staying, communicating and stranger fear^14^. The population is initialized with the help of Eq. (27).

27 $$L_{i}^{0} = {\mathrm{lower}} + r{\mathrm{and}}\left( {0,1} \right) \cdot ({\text{upper - lower}})$$

**Fig. 6:**
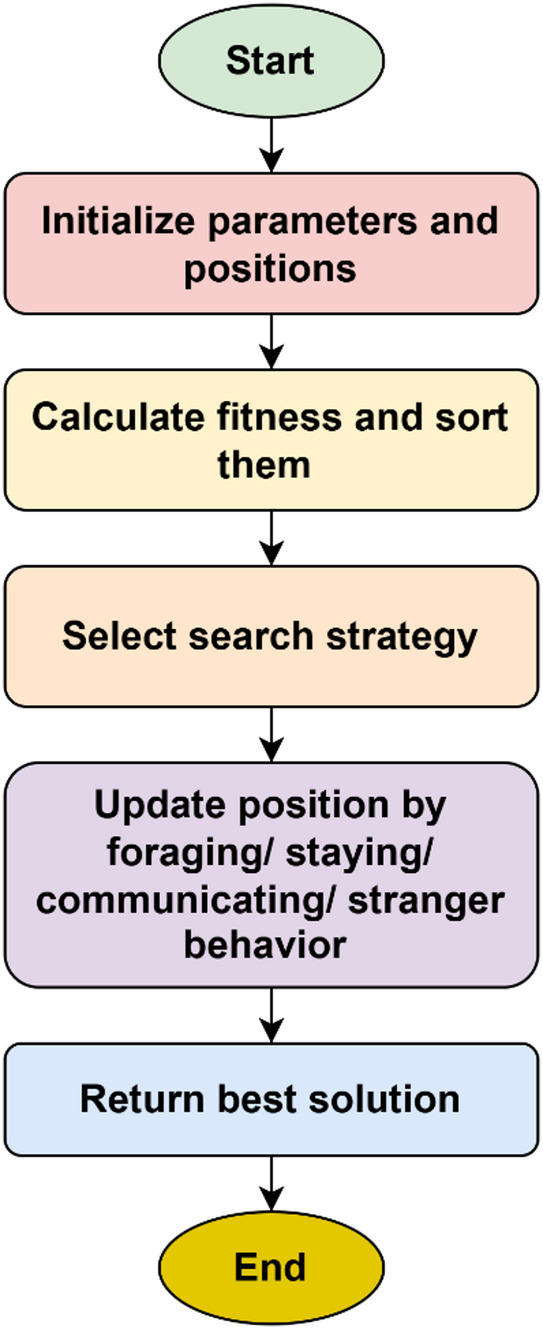
Flow process of parrot optimization.

Lower and upper stand for the lower and upper boundaries of the searching space with a population size of P and maximum iteration limit of ${M}_{j}$. $rand\left(\mathrm{0,1}\right)$ refers to any random number in the range 0 to 1. Figure 6 shows the flow process of parrot optimization.

*Step 1*: Foraging

Foraging is nothing but identifying food source and attaining them in groups or in person considering the location, orientation, position of owner, abundance of food with the help of visual and odor clues. They fly to the food source based upon the below Eq. (28).

28 $$L_{i}^{t + 1} = \left( {L_{i}^{t} - L_{best} } \right) \cdot {\mathrm{Levy}}\left( {{\mathrm{own}}} \right) + r{\mathrm{and}}\left( {0,1} \right) \cdot \left( {1 - \frac{t}{{M_{j} }}} \right)^{{\frac{2t}{{M_{j} }}}} \cdot L_{{{\mathrm{mean}}}}^{t}$$

${L}_{i}^{t}$ is the current location of parrots, ${L}_{i}^{t+ \mathrm{1} }$ is the next location to be moved on and ${L}_{mean}^{t}$ is the average location which is further defined in Eq. (29).

29 $$L_{{{\mathrm{mean}}}}^{t} = \frac{1}{P}\mathop \sum \limits_{k = 1}^{P} L_{k}^{t}$$

$Levy\left(own\right)$ indicates the position in accordance with the owner as described in (30).

30 $${\mathrm{Levy}}\left( {{\mathrm{own}}} \right) = \frac{\mu \cdot \sigma }{{|u|^{\beta } }}$$

31 $$\mu ,u\sim P(0,{\mathrm{own}})$$

$\beta$ takes a constant of value 1.5. $\sigma$ is calculated using (32).

32 $$\sigma = \left( {\frac{{\beta ! \cdot (\sin \frac{\pi \beta }{2})}}{{\left( {\frac{\beta - 1}{2}} \right)!.\beta \cdot 2^{{\frac{\beta - 1}{2}}} }}} \right)^{{\frac{1}{\beta }}}$$

*Step 2*: Staying

Green cheeked parrots are very friendly and hence often fly to their owner and stay with them for a while in a random manner. This mechanism can be figured out in the Eq. (33).

33 $$L_{i}^{t + 1} = \left( {L_{i}^{t} + L_{best} } \right) \cdot {\mathrm{Levy}}\left( {{\mathrm{own}}} \right) + r{\mathrm{and}}\left( {0,1} \right) \cdot B(1,{\mathrm{own}})$$

$B\mathrm{(1,}own\mathrm{)}$ refers to the owner’s body.

*Step 3*: Communication

These animals are very sociable and communicate well among each other and also with the surroundings around them. This communication involves two flying movements namely flying before communication and returning back after communication. These two processes are defined in Eqs. (34) and (35).

34 $$L_{i}^{t + 1} = 0.2*r{\mathrm{and}}\left( {0,1} \right) \cdot \left( {1 - \frac{t}{{M_{j} }}} \right) \cdot \left( {L_{i}^{t} - L_{{{\mathrm{mean}}}} } \right),\,{\mathrm{if}}\,R \le 0.5$$

35 $$L_{i}^{t + 1} = 0.2*r{\mathrm{and}}\left( {0,1} \right) \cdot \exp \left( { - \frac{t}{{r{\mathrm{and}}\left( {0,1} \right).M_{j} }}} \right),\,{\mathrm{if}}\,R > 0.5$$

*R* is a random number between 0 and 1.

*Step 4*: Stranger fear

Any animal is scared of strangers and so are these parrots and hence adopts a sudden movement of flying to seek a safe place and stay away from any potential source of danger. This process can be defined in (36).

36 $$L_{i}^{t + 1} = L_{i}^{t} + r{\mathrm{and}}\left( {0,1} \right) \cdot \cos \left( {0.5\pi \cdot \frac{t}{{M_{j} }}} \right) \cdot (L_{{{\mathrm{best}}}} - L_{i}^{t} )$$

**Fig. 7:**
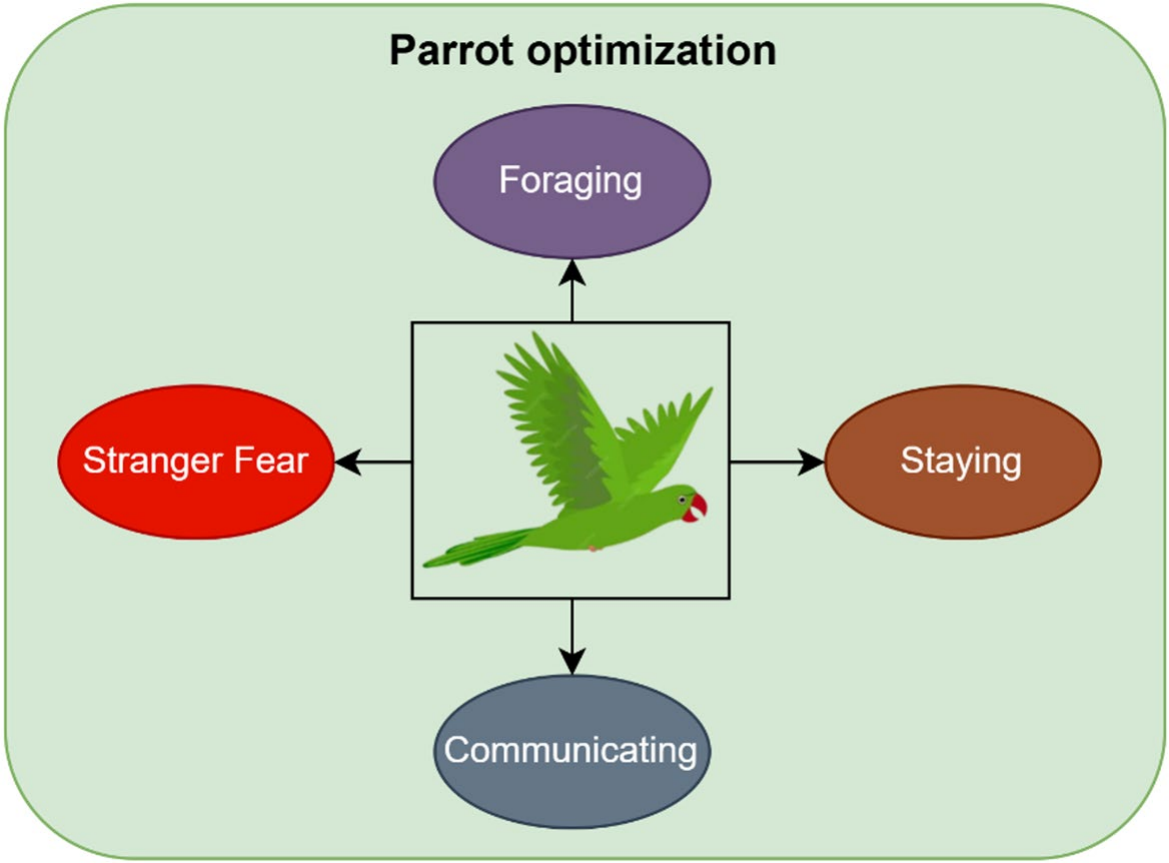
Parrot optimization algorithm.

Figure 7 pictorially represents the parrot optimization algorithm stages.

**Algorithm 1:**
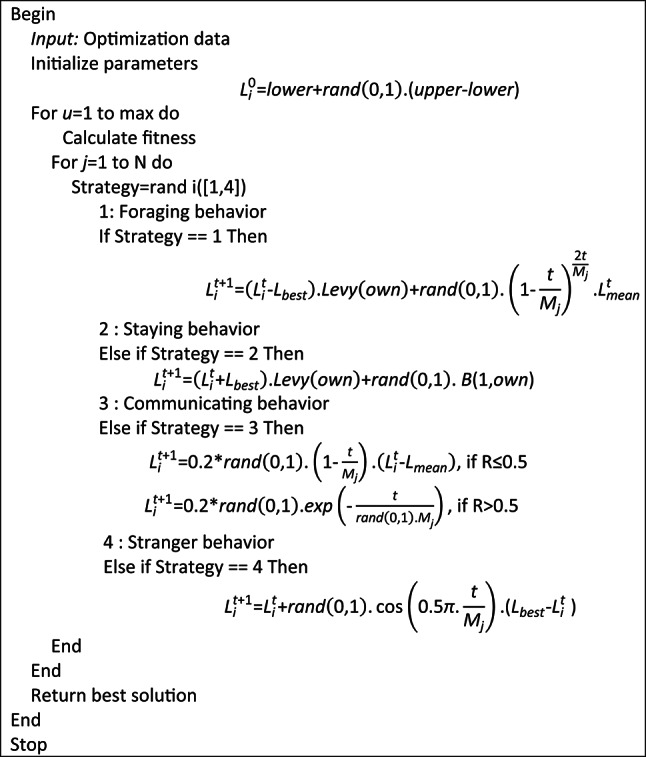
Parrot optimization algorithm.

## Experimental results and analysis

The pancreatic CT image dataset used in this study was carefully preprocessed to ensure consistency and reliability of the results. All images were standardized in terms of size and intensity distribution before further processing. Gabor filtering was applied to enhance texture and edge information while suppressing noise. The dataset obtained from Kaggle consists of labeled images categorized into normal and pancreatic tumour classes, and these labels were retained as provided, assuming standard annotation practices. To address class imbalance, augmentation techniques such as rotation, flipping, scaling, and translation were applied, particularly to the minority class, thereby improving class distribution and model generalization. Further, the use of UNet-based segmentation ensures that only the relevant pancreatic regions are considered for feature extraction and classification, which reduces the impact of background variations and enhances reproducibility. These preprocessing and data handling steps collectively contribute to the robustness and reliability of the proposed framework.

### Experimental setup

The proposed ViT-PO classifier based pancreatic cancer classification model was executed using the latest version of MATLAB R2021b running on a Windows 10 system that had an Intel Core i7 processor with 16 GB RAM. For this process Deep Learning Toolbox, Image Processing Toolbox, and Statistics and Machine Learning Toolbox were utilized from the library. The base model of ViT has 12 layers with a hidden node size of 768, MLP size of 3072, 12 numbers of heads and 86 M parameters. Table 2 presents the hyper parameter details of ViT classifier.

**Table 2:** ViT Hyper parameter details.

S. No	Parameters	Values
1	Patch size	16
2	Number of layers	12
3	Hidden dimensions	768
4	MLP dimension	3072
5	No. of attention heads	12
6	Learning rate	0.001
7	Batch size	32
8	Optimizer	Adam
9	Dropout rate	0.2
10	Epochs	100

The hyperparameters listed in Table 2 are selected based on a combination of standard Vision Transformer (ViT-Base) configurations and empirical tuning to suit the pancreatic CT image dataset. Specifically, parameters such as patch size (16), number of layers (12), hidden dimension (768), MLP dimension (3072), and number of attention heads (12) follow the widely adopted ViT-Base architecture reported in the literature^13^, ensuring a balanced trade-off between model complexity and performance. The training-related parameters, including learning rate (0.001), batch size (32), dropout rate (0.2), and number of epochs (100), are determined through iterative experimentation to achieve stable convergence and avoid overfitting on the given dataset. The Adam optimizer is selected due to its proven efficiency in handling transformer-based models and its ability to adapt learning rates dynamically.

Overall, the parameter configuration ensures compatibility with the ViT architecture, computational feasibility, and optimal performance for the given dataset, while maintaining consistency with established practices in transformer-based medical image analysis.

The hyper parameters of the chosen ViT-Base classifier were carefully picked with utmost care for achieving maximum performance. Patch size of 16 is chosen so that minute details in the image can be well captured, with 12 layers of transformer encoders which represent the depth of the classifier. This value has to be chosen in medium manner as cost and computational complexity increase linearly with the number of encoder blocks. The embedding size was selected as 768 as this is a base model. Higher models such as ViT-Huge and ViT-Large have higher dimensions. The embedding size is also called the hidden dimension of the classifier. It is in this size that the vector is represented for the whole block of transformer encoder.

The dimension of MLP is 3072 which represents the number of hidden neurons that are to be present in the MLP layers for appropriate data learning. 12 number of attention head are present in this classifier for concentrating on various parts of the input images. Batch size was set as 32 for maintaining good balance and to attain model stability. The learning rate was 0.001 as it is the speed at which weights are periodically updated. Since ViTs are critical to optimization, Adam optimizer has been selected for efficiency. The rate of dropout has been rightly set as 0.2. The model has been trained for 100 epochs which provides good speed, convergence, and average computation.

### Execution and results

Implementation of the proposed work was carried out with the help of 1411 images acquired from Pancreatic CT image dataset. In this dataset, 646 normal images and 765 pancreatic tumour images were present. A total of 400 images were created in data augmentation process which were appended to the original dataset of 1411 images thus taking the total count of input images to 1811, respectively. Table 3 below displays the partitioning of input data for training and testing phases. The images were split into a ratio of 70:30 for training and testing phases. 1268 training images and 543 testing images were employed for the execution of the proposed ViT-PO model.

**Table 3:** Input data partition.

	Training images	Testing images	Total
Normal	421	225	646
Pancreatic tumour	847	318	1165
Total	1268	543	1811

**Fig. 8:**
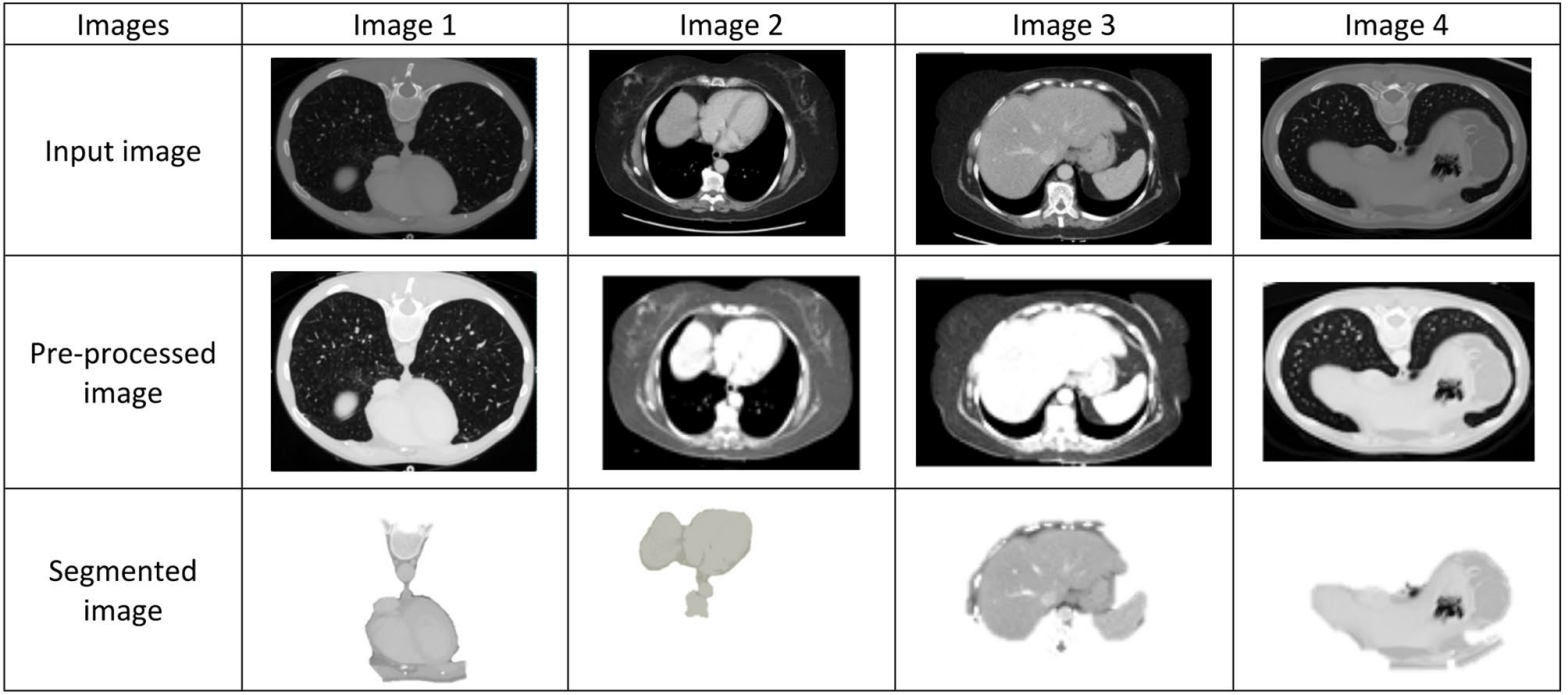
Sample, preprocessed and segmented images.

Figure 8 displays the sample images collected from the Pancreatic CT image dataset in addition to preprocessed images with the help of Gabor filter and UNet based segmented images.

### Performance measures

In this segment, some of the standard performance measures like accuracy, precision, recall, F1-Score and Matthew’s correlation coefficient (MCC) are calculated for assessing the classification performance of the proposed ViT-PO model. The classification outcome will be correct or wrong, which can be signified as one among the four popular classes of true positive (TP), true negative (TN), false positive (FP) and false negative (FN).

TP: Precise classification of positive class.

TN: Precise classification of negative class.

FP: Imprecise classification of positive class.

FN: Imprecise classification of negative class.

Equations for the calculation of accuracy, precision, recall, F1-Score and MCC have been given in (37) to (41).

37 $${\mathrm{Accuracy}} = \frac{{\text{TP + TN}}}{{\text{TP + TN + FP + FN}}}$$

38 $${\mathrm{Precision}} = \frac{{{\mathrm{TP}}}}{{\text{TP + FP}}}$$

39 $${\mathrm{Recall}} = \frac{{{\mathrm{TP}}}}{{\text{TP + FN}}}$$

40 $${\mathrm{F}}_{{1 }} {\mathrm{score}} = \frac{{\mathrm{2*precision*recall}}}{{\text{precision + recall}}}$$

41 $${\text{MCC = }}\frac{{\left( {\text{TP*TN - FP*FN}} \right)}}{{\sqrt {\text{(TP + FP)(TP + FN)(TN + FP)(TN + FN)}} }}$$

**Fig. 9:**
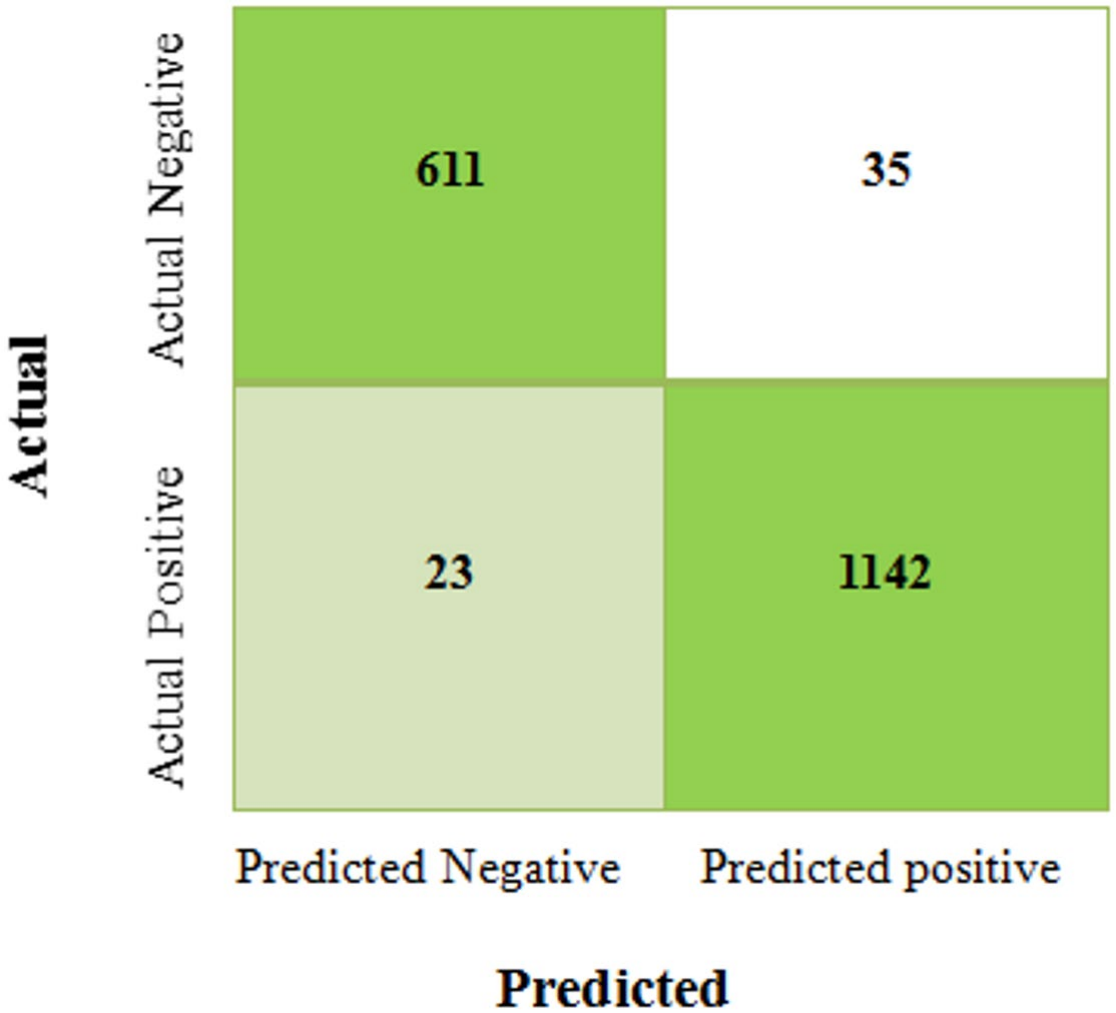
Confusion matrix of the proposed ViT classifier.

Figure 9 displays the confusion matrix produced by the proposed ViT-PO based pancreatic cancer classification system.

The proposed ViT model has achieved a good classification performance with 1142 true positives and 611 true negatives. There were only 23 false negatives and 35 false positives, thus signaling near-perfect balance. These results firmly establish the ViT-PO model’s exceptional precision and trust worthiness in the field of computer-aided diagnosis. Table 4 presents the values achieved by the proposed ViT-PO system in the process of training and testing the pancreatic CT images. The proposed model attained an accuracy of 99%, precision value of 98.5%, recall value of 97.7%, F1-score of 96.4% and MCC score of 97.3%.

**Table 4:** Performance of ViT-PO classifier.

S. No	Metrics	Training values (%)	Testing values (%)
1	Accuracy	99.2	99
2	Precision	99	98.5
3	Recall	98.1	97.7
4	F1-Score	97.3	96.4
5	MCC	97.9	97.3

### Accuracy analysis

In this section, a relative comparison of accuracy values is made among the proposed model and conventional methods which have been employed in the pancreatic carcinoma classification process. Table 5 exhibits the variance in accuracy values attained by the proposed ViT-PO classifier and existing classification systems like Random Forest (RF), Support Vector Machine (SVM), Convolutional Neural Network (CNN), and Deep Belief Network (DBN) at specified epochs of 25, 50, 75 and 100. After the completion of the 25^th^ cycle, the accuracy score of the proposed model reaches 97.9% and after 50^th^, 75^th^ and 100^th^ epochs, they are 98.2%, 98.5% and 99%. It is noteworthy that the accuracy scores have been consistently high throughout the entire training and testing phases, which reflects the stand-alone performance of the proposed model.

**Table 5:** Accuracy assessment.

Epochs	RF (%)	SVM (%)	CNN (%)	DBN (%)	Proposed model (%)
25	96.1	96.8	97.2	97.4	97.9
50	96.3	96.9	97.5	98	98.2
75	97	97.2	97.8	98.1	98.5
100	97.5	98.1	98.4	98.8	99

**Fig. 10:**
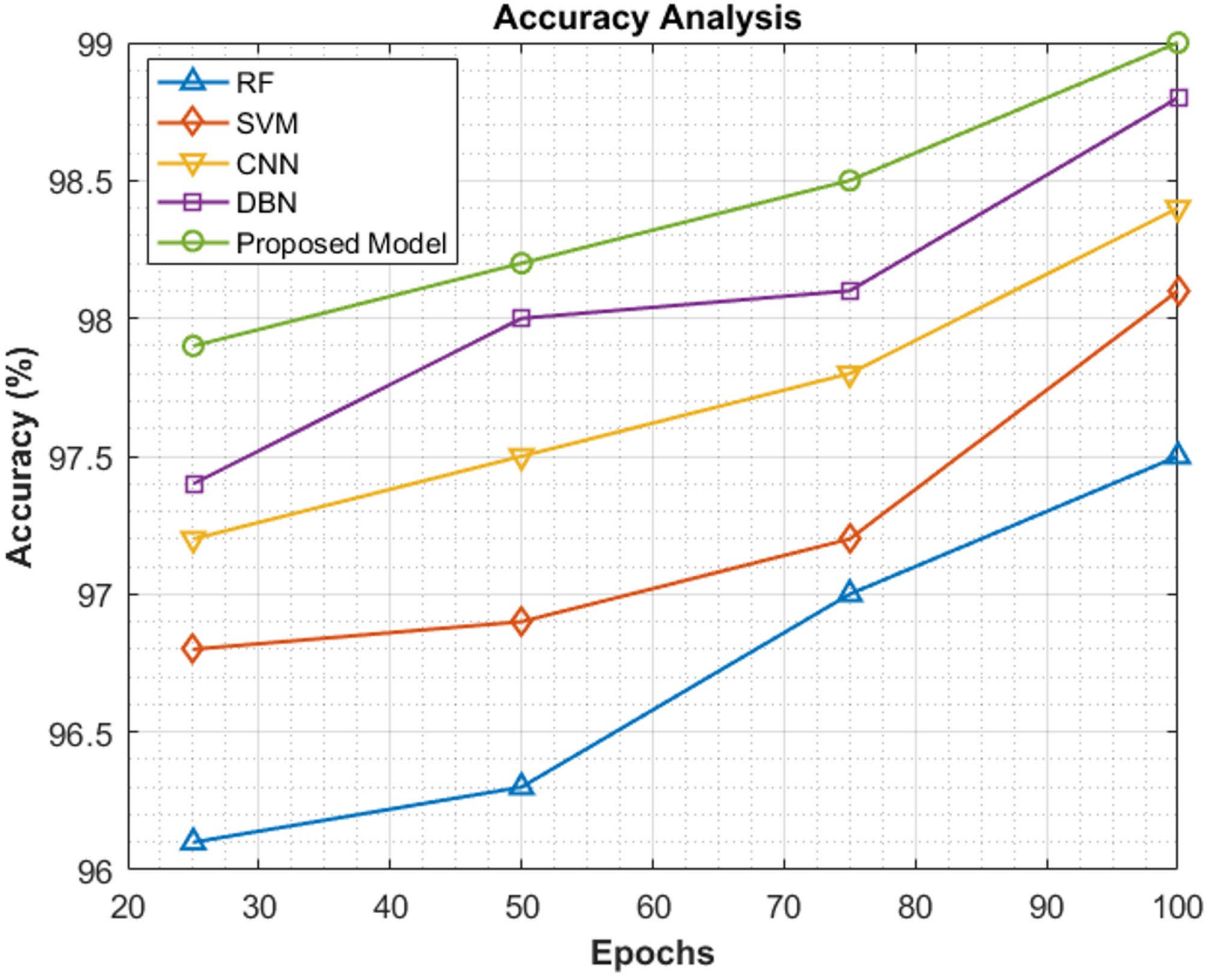
Relative analysis of accuracy.

Figure 10 shows the accuracy of the proposed system in contrast to the accuracy details of other models like RF, CNN, DBN and SVM for highlighting the proposed model’s superiority in the classification of pancreatic carcinoma. The figure is an exact reflection of the excellent performance of the proposed ViT-PO model.

### Precision analysis

This section shows the difference in precision scores among the various classifiers. Table 6 demonstrates the precision values recorded by the proposed ViT-PO model and other existing systems. After reaching 25^th^ and 50^th^ epoch cycles, the precision values attained by the proposed model are 96.2% and 97% correspondingly. Likewise, after 75^th^ and 100^th^ epochs, they reach 97.4% and 98.5% which are fairly high than all of the other models under study thus signifying the exclusiveness of the proposed work.

**Table 6:** Precision analysis.

Epochs	RF (%)	SVM (%)	CNN (%)	DBN (%)	Proposed model (%)
25	93.1	93.6	94.4	95.8	96.2
50	93.9	94.5	95.2	96.3	97
75	94.3	95.6	95.9	96.7	97.4
100	95.8	96.2	96.8	97.3	98.5

**Fig. 11:**
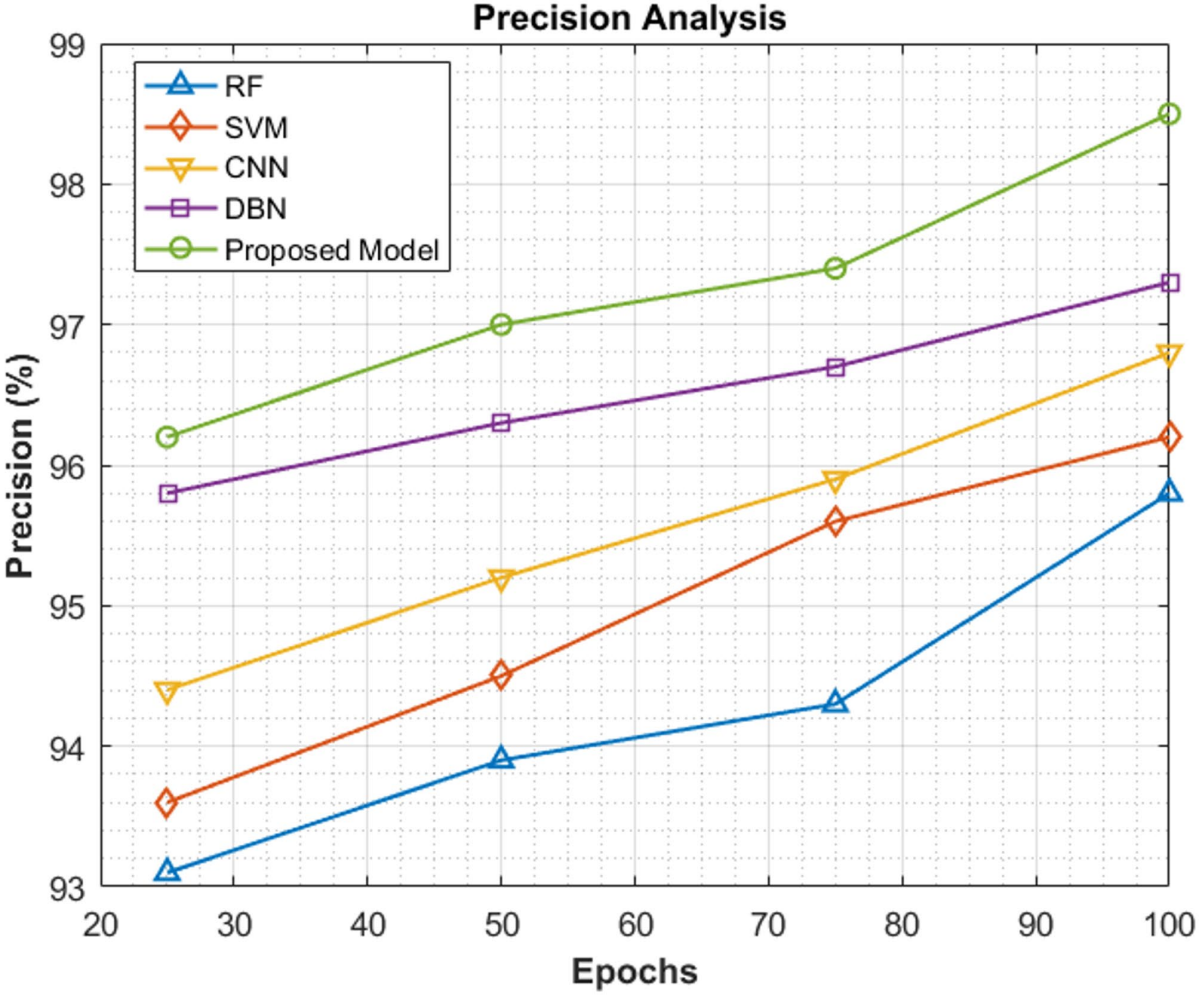
Precision results.

From Fig. 11, which illustrates graphically the above-mentioned precision scores, it is evident that the ViT-PO model works in a better and advanced in terms of precision thus validating the smarter performance of the proposed system.

### Recall analysis

It can be clearly noticed from this section that the proposed model significantly outshines the other models as far as recall scores are concerned. Table 7 contains the different recall scores recorded by the proposed ViT-PO model at four specific epoch cycles of 25, 50, 75 and 100. At the specified epochs, the recall scores are 95.8%, 96.5%, 97.1% and 97.7% respectively, all of which are higher than the existing models explicitly stating the improved performance of the proposed classifier.

**Table 7:** Recall study.

Epochs	RF (%)	SVM (%)	CNN (%)	DBN (%)	Proposed model (%)
25	94.2	94.7	95.1	95.4	95.8
50	94.6	94.9	95.5	96	96.5
75	95.1	95.6	96.3	96.9	97.1
100	95.7	96.4	97	97.4	97.7

**Fig. 12:**
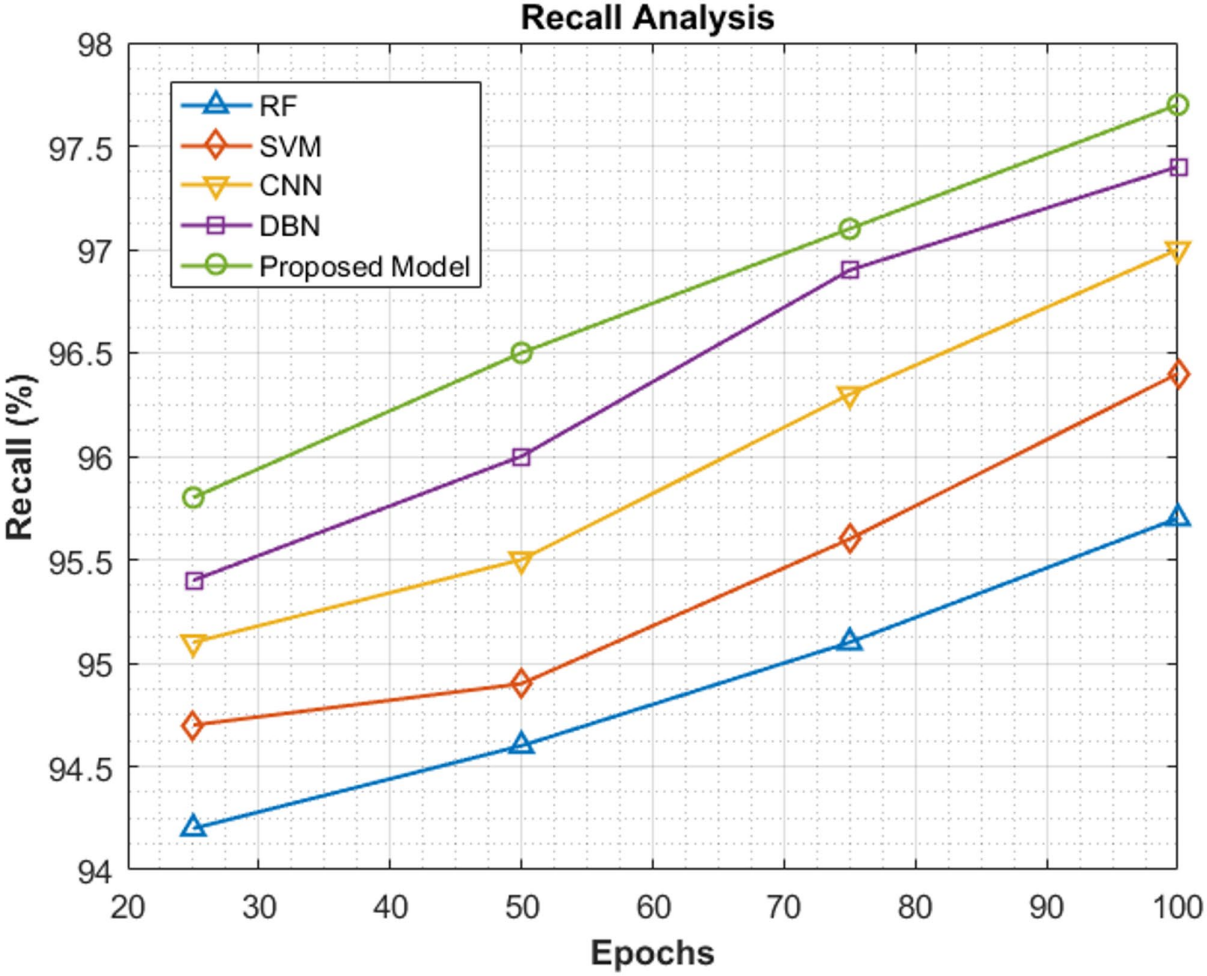
Analysis of recall scores.

The recall values registered at different epoch cycles by existing and proposed classifiers are illustrated in Fig. 12.

### F1-Score analysis

The analysis of F1-Score done in this section clearly shows that the proposed work of ViT-PO attains much better F1-Score than all the preceding models in the classification of pancreatic carcinoma. Table 8 consists of the F1 scores achieved by the proposed model and existing models at 25^th^, 50^th^, 75^th^ and 100^th^ epochs. The F1-Scores achieved by the proposed method were 95.3%, 95.8%, 96.1% and 96.4% at the mentioned epochs.

**Table 8:** Comparative analysis of F1-score.

Epochs	RF (%)	SVM (%)	CNN (%)	DBN (%)	Proposed model (%)
25	93.7	94.1	94.6	95.1	95.3
50	93.9	94	94.2	95.5	95.8
75	94.4	94.8	95.3	95.9	96.1
100	95	95.2	95.7	96.1	96.4

**Fig. 13:**
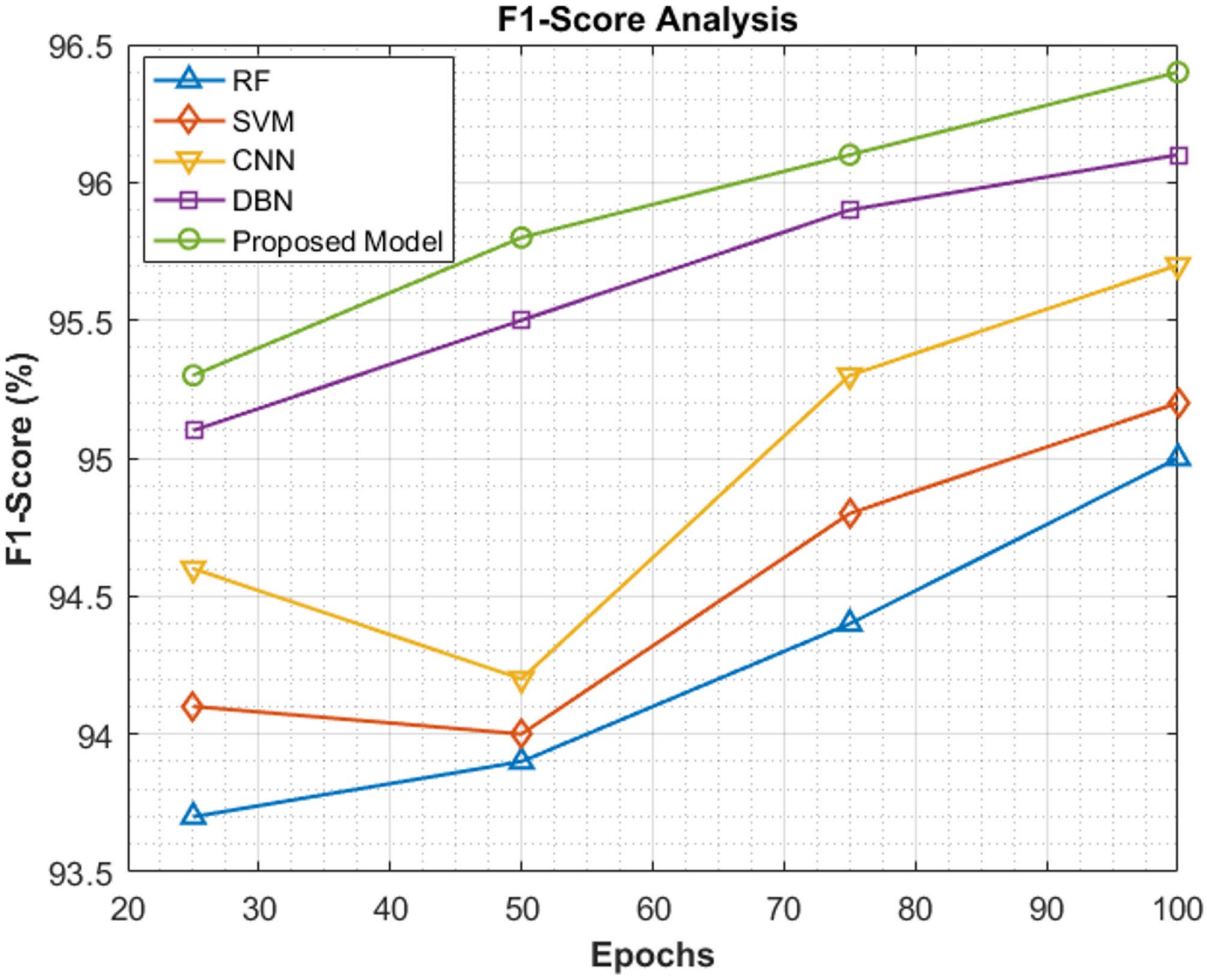
F1-score comparison.

Figure 13 depicts the F1-Score comparison in a graphical manner which was developed from the above table. Consistent good performance by the proposed model in terms of F1-Score increases the reliability of the model hugely.

### MCC analysis

A comparison is made between the current classifiers and the proposed one regarding the MCC scores. Table 9 describes the MCC values produced by the proposed ViT-PO classifier after completing certain epochs of 25, 50, 75 and 100. The MCC score at 25^th^ epoch is 95.9%, 50^th^ epoch is 96.2%, 75^th^ epoch is 96.8% and after 100^th^ epoch, it reaches 97.3% finally. The MCC scores of the proposed model surpass that of the other conventional models in all the specified epoch cycles portraying the robustness of the proposed ViT-PO model.

**Table 9:** MCC scores.

Epochs	RF (%)	SVM (%)	CNN (%)	DBN (%)	Proposed model (%)
25	94	94.5	95.3	95.6	95.9
50	94.2	94.8	95.4	95.7	96.2
75	94.7	95	95.7	96.1	96.8
100	95.1	95.4	96	96.8	97.3

**Fig. 14:**
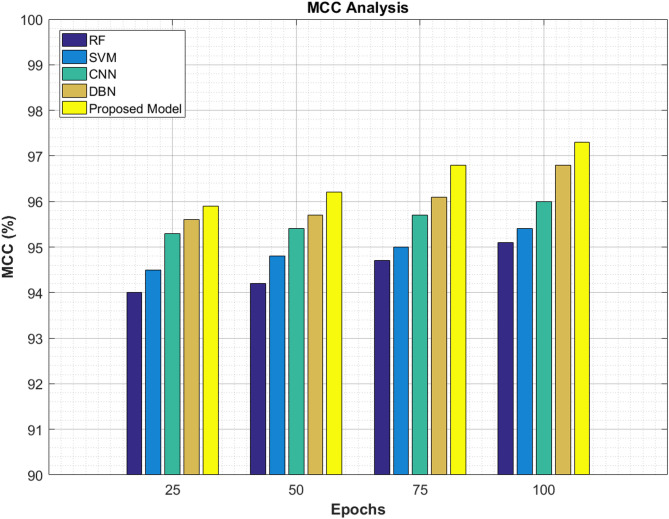
MCC analysis.

Figure 14 presents a mathematical graphical model of MCC scores achieved by different classifiers at selected epochs.

### TPR analysis

**Fig. 15:**
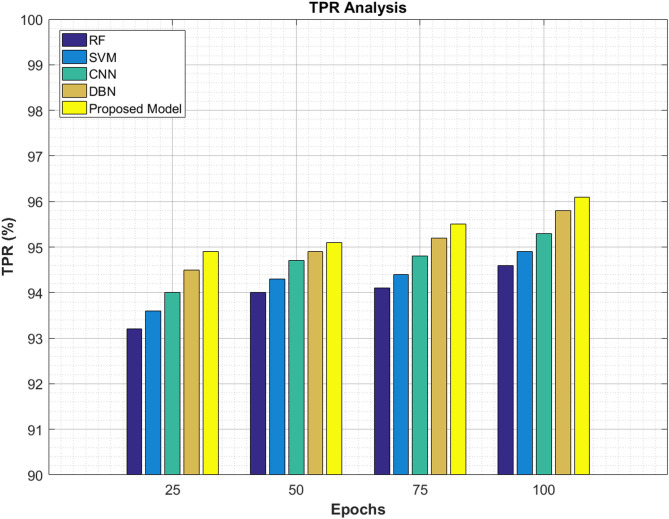
TPR graphical analysis.

This part carries the true positive rate (TPR) analysis for pancreatic cancer classification using ViT-PO model. The results are given in Table 10 and Fig. 15. The proposed method attains the highest TPR in all the epoch iterations, insisting the advanced sensitivity it has in detecting true positives cases which is very essential for medical diagnosis. The proposed model attain 94.9% and 95.1% in the initial epochs and finally reach 95.5% and 96.1% at the end of 100 epochs which are comparatively higher than all the existing conventional methods.

**Table 10:** TPR analysis.

Epochs	RF (%)	SVM (%)	CNN (%)	DBN (%)	Proposed model (%)
25	93.2	93.6	94	94.5	94.9
50	94	94.3	94.7	94.9	95.1
75	94.1	94.4	94.8	95.2	95.5
100	94.6	94.9	95.3	95.8	96.1

#### FPR analysis

**Fig. 16:**
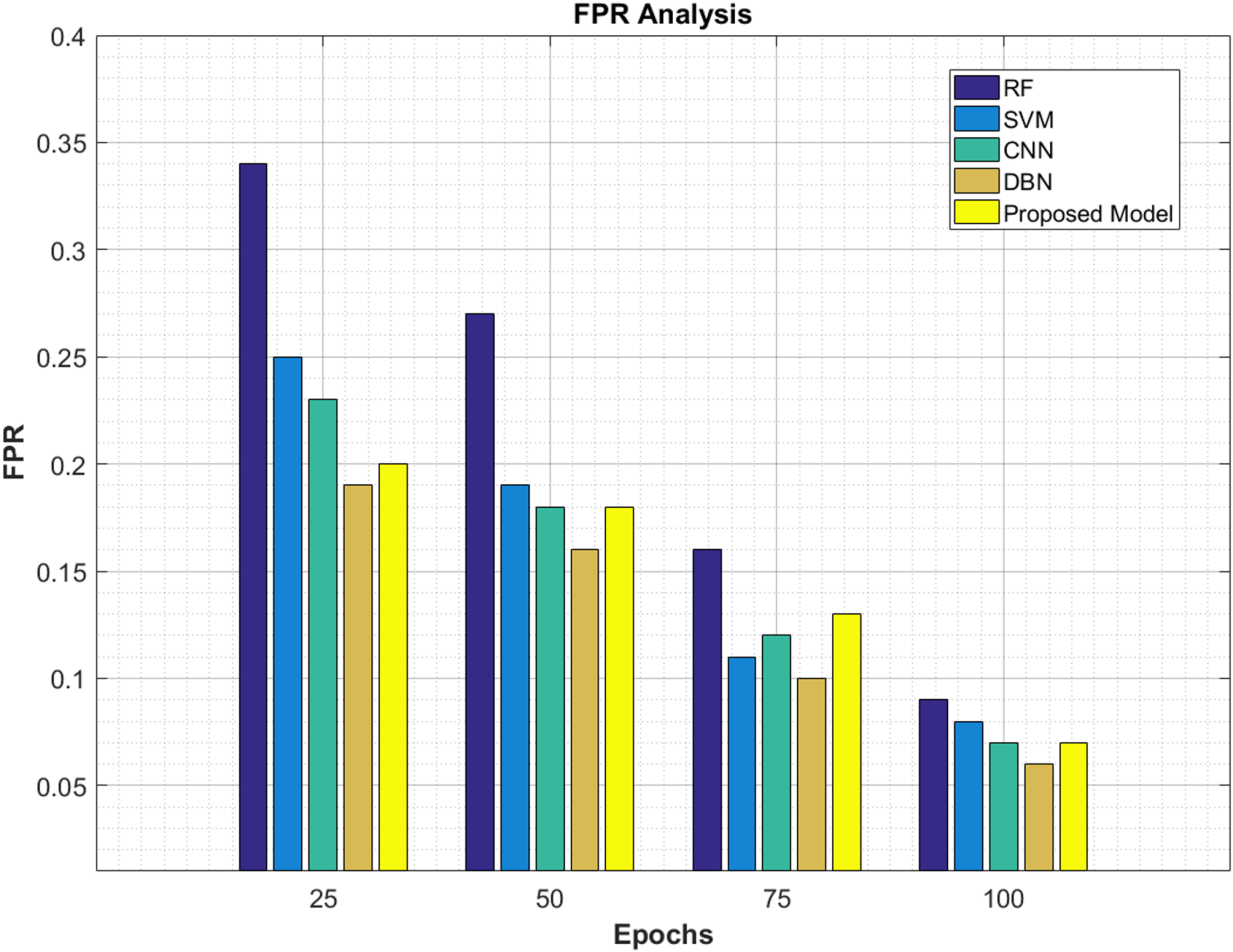
FPR analysis.

The False Positive Rate (FPR) Analysis has been presented here between the current approaches and the proposed model as stated in Table 11 and Fig. 16. It is apparent from the given table that the proposed ViT-PO method possesses the lowest rate of FPR, exhibiting a better proficiency to mitigate false cases thus aiding oncologists in their work. The proposed work is superior to all the other compared models of RF, SVM, CNN and DBN.

**Table 11:** FPR analysis.

Epochs	RF (%)	SVM (%)	CNN (%)	DBN (%)	Proposed model (%)
25	0.34	0.25	0.23	0.19	0.20
50	0.27	0.19	0.18	0.16	0.18
75	0.16	0.11	0.12	0.10	0.13
100	0.09	0.08	0.07	0.06	0.07

Table 12 provides the consolidated scores of all the classifiers under study in the final epoch cycle of 100. In all the areas of evaluation, the proposed ViT-PO model surpasses all the classifiers at least by a small margin, indicating the phenomenal performance.

**Table 12:** Consolidated performance in final epoch cycle.

Method	RF	SVM	CNN	DBN	Proposed model
Accuracy (%)	97.5	98.1	98.4	98.8	99
Precision (%)	95.8	96.2	96.8	97.3	98.5
Recall (%)	95.7	96.4	97	97.4	97.7
F1-Score (%)	95	95.2	95.7	96.1	96.4
MCC (%)	95.1	95.4	96	96.8	97.3
TPR (%)	94.6	94.9	95.3	95.8	96.1

**Fig. 17:**
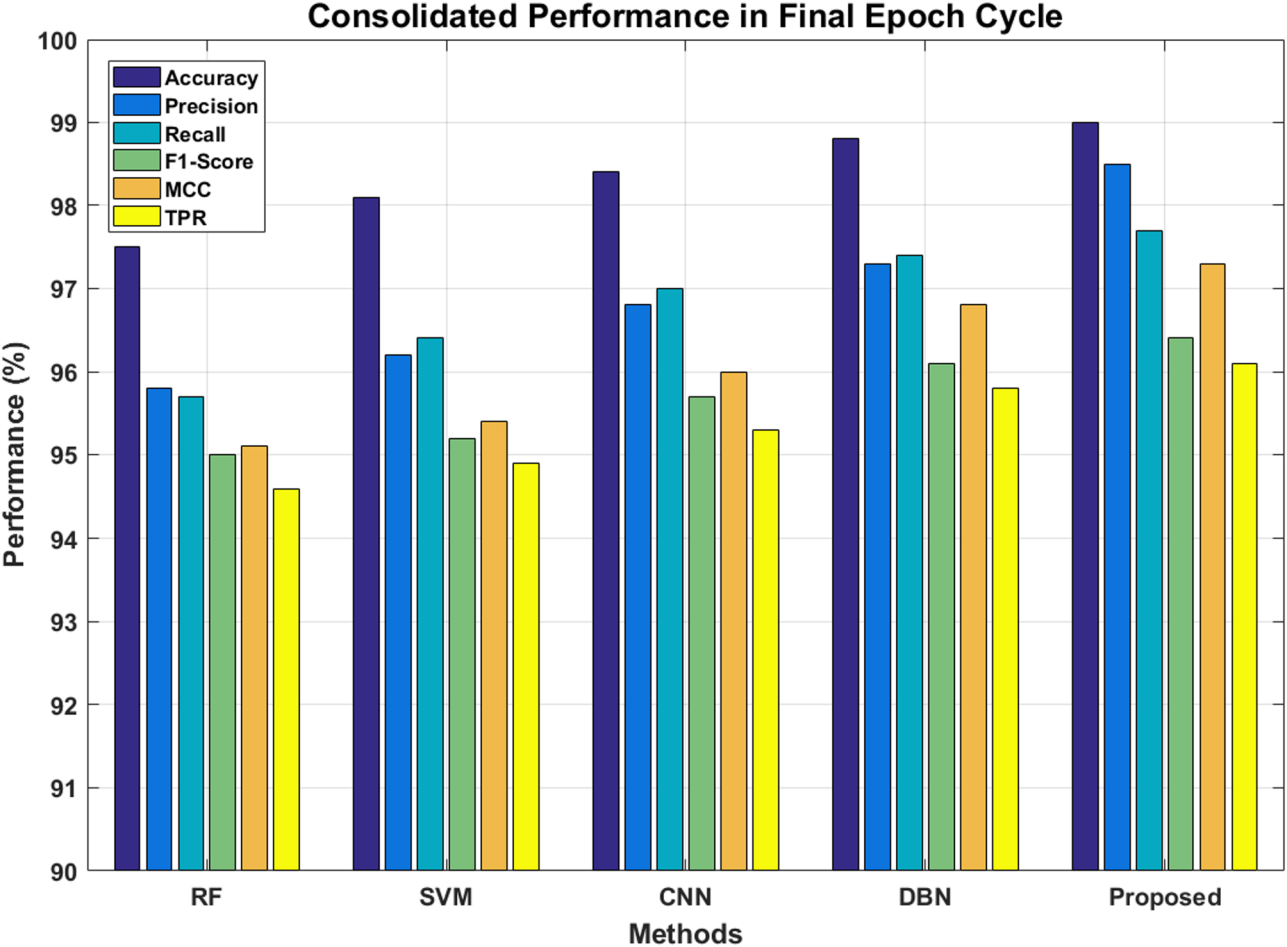
Consolidated performance graph.

The same has been visually shown in Fig. 17 to provide a wholesome view.

#### Optimization analysis

This section shows the analysis of different optimization algorithms in the process of pancreatic cancer classification. The comparative evaluation is done with the proposed Parrot optimization algorithm against standard optimization algorithms like genetic algorithm, Harris Hawk optimization and Red Deer optimization in terms of accuracy values as listed in Table 13. Notably, the proposed optimization algorithm attains a higher accuracy value in the process of same pancreatic cancer using different datasets. This endorses the effectiveness of the proposed ViT-PO model in fine-tuning parameters for achieving exceeded performance.

**Table 13:** Comparison of optimization algorithms.

Optimizer	Accuracy (%)
Genetic algorithm^25^	93.9
Harris Hawk optimization^26^	96
Red deer optimization^27^	98.51
Proposed parrot optimization	99

#### Misclassification analysis

This segment consists of an investigation about the misclassifications and errors that happened in the process of pancreatic cancer classification. Although the proposed model performed well, there were certain misclassifications. The reason for this can be attributed to the fact that some of the images were ambiguous and not enough details could be gathered from it. Another setback was due to improper lighting during the process of capturing the image. Normal images were misclassified as pancreatic tumour due to the presence of minor artifacts. This study could offer an understanding of the mistakes that happened, where they happened and what went wrong in the whole process. These details will help in identifying and avoiding those particular mistakes in future. It can also contribute well to augmenting the classification accuracy and upgrade the overall performance of the proposed work.

#### Convergence and stability analysis of POA

To evaluate the convergence behavior and stability of the Parrot Optimization Algorithm (POA), experiments were conducted across multiple independent runs. The fitness value (classification accuracy) was monitored over iterations, and statistical consistency was analyzed (Table 14).

**Table 14:** Convergence analysis of POA.

Iterations	Accuracy (%)
10	97.8
20	98.3
30	98.7
40	98.9
50	99.0

The convergence analysis shows that the POA rapidly improves classification performance within the first few iterations and stabilizes after approximately 40–50 iterations, indicating efficient convergence without oscillatory behavior.

The stability analysis across multiple runs demonstrates minimal variation in performance metrics, confirming that the optimization process is consistent and does not suffer from instability. The negligible difference in accuracy (within ± 0.2%) across runs validates the robustness of POA in refining classification outcomes (Table 15).

**Table 15:** Stability analysis over multiple runs.

Run No	Accuracy (%)	Precision (%)	Recall (%)	F1-Score (%)	MCC (%)
Run 1	99.0	98.5	97.7	96.4	97.3
Run 2	98.9	98.3	97.5	96.2	97.1
Run 3	99.0	98.4	97.6	96.3	97.2
Run 4	98.8	98.2	97.4	96.1	97.0
Run 5	99.0	98.5	97.7	96.4	97.3

These results confirm that POA achieves a balanced exploration–exploitation trade-off, ensuring both fast convergence and stable performance in the proposed framework.

#### Computational analysis

Integrating segmentation, transformer-based classification, and optimization introduces additional computational overhead. However, the framework is designed in a sequential and modular manner, where each component operates on progressively refined data, thereby reducing unnecessary computation. Specifically, UNet-based segmentation restricts processing to relevant regions, which reduces input complexity for subsequent stages, and YOLOv11 extracts compact feature representations instead of operating on full-resolution images. Furthermore, the Vision Transformer used corresponds to a base configuration, and training parameters are carefully controlled to maintain computational feasibility. The optimization stage (POA) is applied only as a lightweight refinement step rather than during full network training, limiting its computational impact.

Apart from the theoretical point of view, real-world implementation of the proposed work in medical arena needs a certain degree of computational findings. In order to review the practical implications of the proposed ViT-PO model, its computational efficiency was evaluated and. The proposed ViT-PO required 108 s for execution, with almost 500 MB memory space.

#### Cross-validation analysis

To further strengthen the reliability of the proposed model, a fivefold cross-validation experiment was conducted on the same dataset. The dataset was randomly partitioned into five equal subsets, where in each iteration, four subsets were used for training and one subset was used for testing. The average performance across all folds is reported in Table 16.

**Table 16:** Cross-validation results of proposed ViT-PO model.

Fold	Accuracy (%)	Precision (%)	Recall (%)	F1-Score (%)	MCC (%)
Fold 1	98.8	98.2	97.5	96.2	97.0
Fold 2	99.0	98.5	97.7	96.4	97.3
Fold 3	98.9	98.3	97.6	96.3	97.1
Fold 4	99.1	98.6	97.8	96.5	97.4
Fold 5	98.9	98.4	97.6	96.3	97.2
Average	98.94	98.40	97.64	96.34	97.20

The cross-validation results demonstrate consistent performance of the proposed ViT-PO model across different data splits, with minimal variation in evaluation metrics. The average accuracy of 98.94% closely aligns with the previously reported results, indicating that the model does not overfit to a specific train-test split. The low standard deviation across folds confirms the stability and generalization capability of the proposed framework.

Although external dataset validation is not included due to dataset availability constraints, the cross-validation analysis provides strong evidence of the robustness and reliability of the proposed model. Future work will focus on validating the model on larger and multi-institutional datasets to further enhance its clinical applicability.

#### Ablation analysis

To evaluate the contribution of each component in the proposed framework, an ablation study is conducted by progressively enabling different modules of the system. The performance is measured using standard evaluation metrics on the same dataset and experimental setup.

The ablation results in Table 17 clearly demonstrate the contribution of each component in the proposed framework. The baseline CNN model shows comparatively lower performance due to limited feature representation capability. The use of Vision Transformer improves performance by capturing global contextual dependencies. Incorporating UNet-based segmentation further enhances the results by focusing on relevant pancreatic regions. The addition of YOLOv11-based feature extraction improves multi-scale feature representation, leading to better classification outcomes. Finally, the inclusion of Parrot Optimization results in the highest performance, indicating its effectiveness in refining classification decisions. Overall, the progressive improvement across configurations validates that each module contributes significantly to the final performance of the proposed ViT-PO framework.

**Table 17:** Ablation study of proposed ViT-PO framework.

Model variant	Accuracy (%)	Precision (%)	Recall (%)	F1-Score (%)	MCC (%)
Baseline CNN	96.8	95.9	95.4	95.6	94.8
ViT only	97.5	96.8	96.2	96.4	95.9
UNet + ViT	98.1	97.4	96.9	97.1	96.5
UNet + YOLOv11 + ViT	98.6	97.9	97.3	97.5	96.9
Full MODEL (ViT-PO)	99.0	98.5	97.7	96.4	97.3

#### Discussion

The proposed ViT-PO framework demonstrates strong and consistent performance across all key evaluation metrics, indicating its effectiveness for pancreatic cancer classification. The model achieves an overall accuracy of 99%, with precision of 98.5%, recall of 97.7%, F1-score of 96.4%, and MCC of 97.3%, reflecting a well-balanced classification capability. The high MCC value, in particular, confirms the robustness of the model in handling both positive and negative classes without bias, which is critical in medical diagnosis.

From a diagnostic perspective, the model attains a true positive rate of 96.1% and a low false positive rate of 0.07%, indicating high sensitivity in detecting tumour cases while minimizing false alarms. This is clinically significant, as reducing false negatives is essential to avoid missed diagnoses, and controlling false positives prevents unnecessary follow-up procedures.

The ablation analysis further validates the contribution of each component in the proposed framework. The performance improves progressively from 96.8% (baseline) to 99% (full model), confirming that segmentation (UNet), feature extraction (YOLOv11), transformer-based classification (ViT), and optimization (POA) each play a meaningful role in enhancing the overall system. Additionally, the cross-validation results show an average accuracy of 98.94%, demonstrating the model’s stability and generalization capability across different data splits.

The optimization analysis reveals that the proposed POA achieves higher accuracy compared to other algorithms such as Genetic Algorithm (93.9%), Harris Hawk Optimization (96%), and Red Deer Optimization (98.51%), confirming its effectiveness in refining classification outcomes. Convergence analysis indicates stable performance within 40–50 iterations, and multi-run experiments show negligible variation (± 0.2%), validating its reliability.

From a clinical standpoint, the integration of segmentation-based region focusing and attention-based global learning enables the model to capture both anatomical structures and contextual dependencies. This makes the system suitable as a decision-support tool for radiologists, assisting in early-stage detection and improving diagnostic consistency. However, the absence of external dataset validation and reliance on binary classification limit immediate clinical deployment.

Overall, the results indicate that the proposed framework provides a highly accurate, stable, and clinically relevant solution, while highlighting the need for further validation on diverse datasets and real-world clinical settings.

## Conclusion

The incidence of pancreatic carcinoma has shown a consistent rise over recent years, coupled with persistently low survival rates due to late-stage diagnosis. This highlights the critical need for reliable and automated diagnostic systems capable of supporting early detection. In this context, the proposed work presents a structured and integrated framework for pancreatic cancer classification using CT images, combining Gabor-based preprocessing, UNet-driven segmentation, YOLOv11-based feature extraction, and Vision Transformer classification enhanced through Parrot Optimization. This multi-stage design enables effective capture of both local structural characteristics and global contextual dependencies, thereby strengthening the overall diagnostic capability of the system. The experimental findings demonstrate that the proposed ViT-PO model achieves high performance across multiple evaluation metrics, including accuracy (99%), precision (98.5%), recall (97.7%), F1-score (96.4%), and MCC (97.3%), along with a high true positive rate and minimal false positive rate. These results indicate not only strong classification capability but also consistency and reliability, which are essential in medical diagnosis. Further, comparative analysis confirms that the proposed model outperforms conventional classifiers such as Random Forest, CNN, DBN, and SVM, thereby validating the effectiveness of the integrated approach. The strength of the proposed framework lies in its ability to combine segmentation-based region focusing, detection-driven feature extraction, transformer-based global learning, and metaheuristic optimization within a single pipeline. This coordinated design directly addresses key limitations observed in existing methods and contributes to improved diagnostic performance. However, the current model is limited to binary classification and involves moderate computational complexity, which may impact large-scale deployment. Future work can focus on extending the model for multi-class classification of pancreatic disease stages, optimizing computational efficiency, and validating the framework on larger and multi-institutional datasets. Integration with additional imaging modalities and clinical parameters can further enhance its applicability. Overall, the proposed framework offers a promising and reliable approach for computer-aided pancreatic cancer diagnosis, with potential to support early detection and improve clinical decision-making. The proposed framework, while demonstrating strong performance, is currently limited by the use of a moderately sized dataset and binary classification, which may affect its generalizability across diverse clinical scenarios. Additionally, the computational complexity associated with transformer-based models may pose challenges for real-time deployment in resource-constrained settings. From a clinical perspective, the model can serve as a supportive diagnostic tool to assist radiologists in early detection; however, extensive validation on multi-institutional datasets and integration with clinical workflows are essential before practical adoption.

## Data Availability

The dataset utilized for the execution of the proposed pancreatic carcinoma classification system is available in the following link. https://www.kaggle.com/datasets/jayaprakashpondy/pancreatic-ct-images.
